# Rationalized design of hyperbranched trans-scale graphene arrays for enduring high-energy lithium metal batteries

**DOI:** 10.1126/sciadv.adc9961

**Published:** 2022-08-24

**Authors:** Ruopian Fang, Zhaojun Han, Jibiao Li, Zhichun Yu, Jian Pan, Soshan Cheong, Richard D. Tilley, Francisco Trujillo, Da-Wei Wang

**Affiliations:** ^1^ School of Chemical Engineering, The University of New South Wales, Sydney, NSW 2052, Australia.; ^2^ CSIRO Manufacturing, Lindfield, NSW 2070, Australia.; ^3^ College of Materials Science and Engineering, Yangtze Normal University, Chongqing 408100, China.; ^4^ College of Materials Science and Engineering, Chongqing University, Chongqing 400044, China.; ^5^ Mark Wainwright Analytical Centre, The University of New South Wales, Sydney, NSW 2052, Australia.; ^6^ School of Chemistry, The University of New South Wales, Sydney, NSW 2052, Australia.

## Abstract

Lithium (Li) metal anode have shown exceptional potential for high-energy batteries. However, practical cell-level energy density of Li metal batteries is usually limited by the low areal capacity (<3 mAh cm^−2^) because of the accelerated degradation of high–areal capacity Li metal anodes upon cycling. Here, we report the design of hyperbranched vertical arrays of defective graphene for enduring deep Li cycling at practical levels of areal capacity (>6 mAh cm^−2^). Such atomic-to-macroscopic trans-scale design is rationalized by quantifying the degradation dynamics of Li metal anodes. High-energy Li metal cells are prototyped under realistic conditions with high cathode capacity (>4 mAh cm^−2^), low negative-to-positive electrode capacity ratio (1:1), and low electrolyte-to-capacity ratio (5 g Ah^−1^), which shed light on a promising move toward practical Li metal batteries.

## Teaser

A trans-scale design rationale is proposed for composite Li anodes to achieve high-energy lithium metal batteries.

## INTRODUCTION

The ever-increasing demand for dense energy storage technologies has been driven by the penetration of consumer electronics, electric vehicles, and renewable power stations (*1*). Lithium metal batteries (LMBs) using lithium (Li) metal anodes are believed the future of high-energy battery technology (*2*, *3*), as Li metal has extraordinary theoretical specific capacity (3860 mAh g^−1^) and the most negative standard electrode potential (−3.04 V versus standard hydrogen electrode) (*4*). Despite the high specific capacity of Li metal, boosting the cell-level specific energy (*E*_sp_, in watt-hours per kilogram) of LMBs necessitates the maximization of areal capacity (*C*_areal_, in milliampere-hours per square centimeter), as defined by

$$E_{\text{sp}}=C_{\text{areal}}\times U/M_{\text{cell}}$$

where *U* is the cell voltage (volts) and *M*_cell_ is the cell areal density (grams per square centimeter) inclusive of active and passive components. Maximizing *C*_areal_ corresponds to increasing the portion of electroactive material that determines *E*_sp_ (*5*). Apparently, low *C*_areal_ of Li metal anodes will limit the *E*_sp_ of LMBs.

Development of high–areal capacity Li metal anodes has to surmount several problems. The high reactivity of Li metal induces uncontrolled physicochemical reactions that lead to unstable solid-electrolyte interphase (SEI), nonuniform Li deposition and stripping, and inactive Li accumulation (*6*–*8*). These detrimental effects are amplified at high Li plating/stripping capacity, resulting in the accelerated degradation of Li metal anodes (*9*). Although stable Li metal anodes have been reported, most are restricted to low areal capacity (<3 mAh cm^−2^) (*10*–*13*). Such shallow deposition and stripping of Li metal anodes can hardly be translated to practical high-energy batteries (*2*). LMBs are estimated to deliver a specific energy of 350 Wh kg^−1^ at single-cell level only with areal capacity ≥ 4 mAh cm^−2^ (*14*–*16*). Therefore, discovering high–areal capacity Li metal anodes performing at high reversibility and enduring cycling is the unresolved priority challenge for LMBs.

In this work, we systematically investigate the degradation dynamics of Li metal anodes and identify a key descriptor that correlates the durability of high–areal capacity Li metal anodes with the design rationale of Li host structures. We determine that an ideal Li host structure requires both macroscopic percolating conductive network to circumvent impedance buildup and well-engineered atomic-to-microsocpic structures to induce spatially homogeneous Li plating/stripping with high reversibility. Accordingly, we propose the design of hyperbranched vertical arrays of defective graphene (HVDG) for enduring deep Li deposition and stripping at practical levels of areal capacity (>6 mAh cm^−2^). The highly dispersed defects and vertical cavities of graphene arrays enable well-regulated Li cycling behavior with high reversibility. The hyperbranched architecture facilitates high-efficiency charge transfer that counts for the enduring Li cycling. As a result, a prototype energy-dense LMB cell equipped with the Li-HVDG anode demonstrates stable cyclability under realistic conditions with high areal capacity, low negative-to-positive electrode capacity (N/P) ratio, and lean electrolyte supply.

## RESULTS

### Quantifying the degradation dynamics during Li cycling

**Fig. 1.**
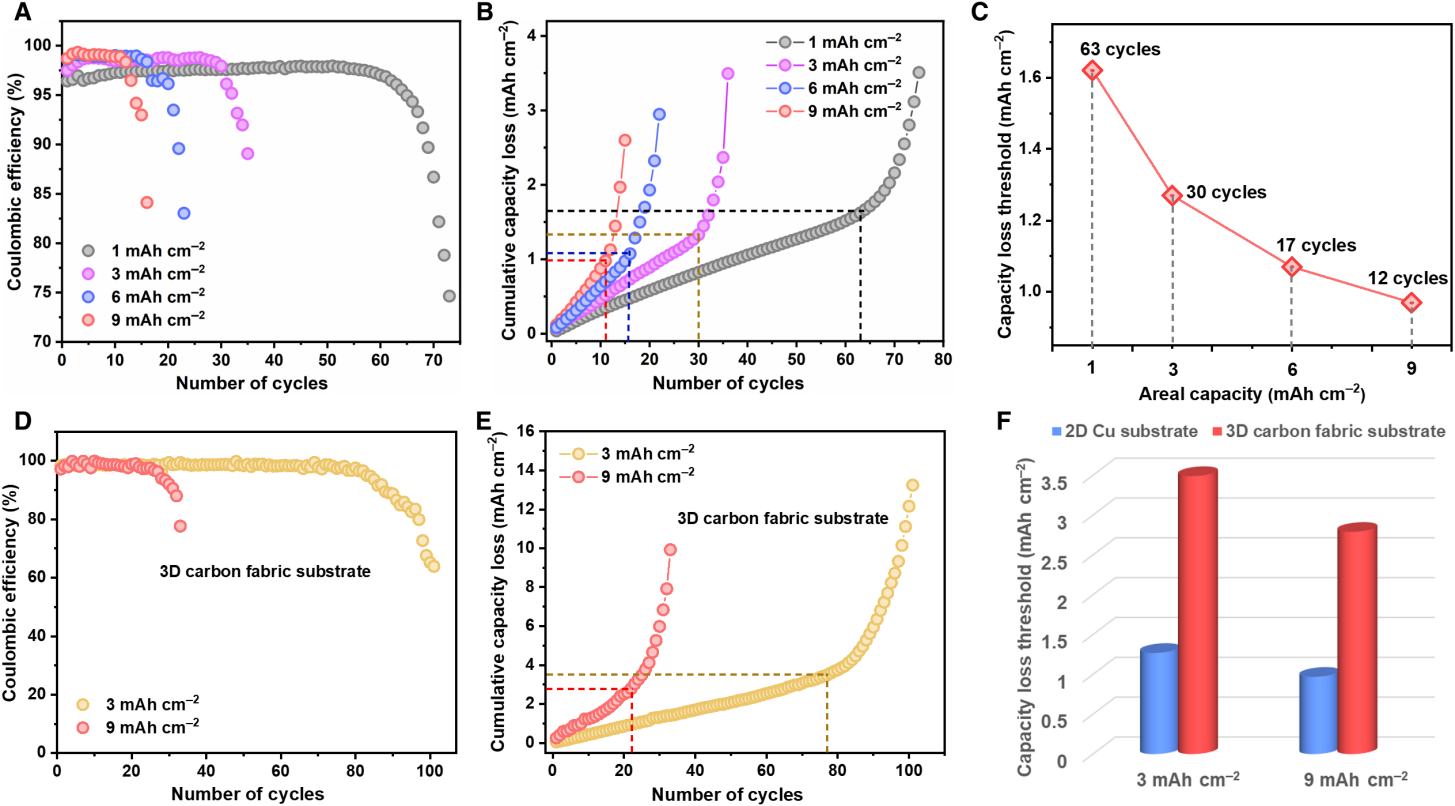
Quantitative analysis of Li metal cycling on planar and 3D substrates. (**A**) Coulombic efficiency (CE) of Li||Cu cells under areal capacities of 1, 3, 6, and 9 mAh cm^−2^ at 1 mA cm^−2^. (**B**) Cumulative capacity loss (*C*_loss_) as a function of number of cycles in Li||Cu cells. The dashed lines mark the number of stable cycles (vertical line) and capacity loss threshold (horizontal line) that highlights the onset of accelerated degradation. The stable cycles are defined where the CE drops below 97% for the cells cycled at 1 mAh cm^−2^ and below 98% for the cells cycled from 3 to 9 mAh cm^−2^. (**C**) Capacity loss threshold (*C*_loss threshold_) as a function of areal capacity of Li||Cu cells, marked with the corresponding numbers of stable cycles. (**D**) CE of Li||3D CF cells under areal capacities of 3 and 9 mAh cm^−2^. (**E**) Cumulative capacity loss as a function of number of cycles in Li||3D CF cells. (**F**) Comparison of capacity loss threshold in Li||Cu cells and Li||3D CF cells at 3 and 9 mAh cm^−2^.

Li||Cu half cells were used to investigate the degradation dynamics of Li metal anodes as a function of areal capacity, as Cu is the most common substrate for Li cycling (*17*). As displayed in Fig. 1A, the cycling stability of Li||Cu cells decreases with increasing areal capacity, despite the slightly increasing trend of Coulombic efficiency (CE) in the initial cycles (fig. S1). Such rapidly degrading performance with increasing areal capacity highlights the dilemma between high areal capacity and long life span of Li metal anodes.

We further analyzed the cumulative capacity loss (*C*_loss_) on the Cu substrate upon cycling to investigate the underlying cause of limited life span at high areal capacity. As Coulombic inefficiency (1 − CE) corresponds to capacity loss between the plating and stripping processes (*7*), *C*_loss_ over cycling is calculated on the basis of (1 − CE) and areal capacity (*C*)

$$C_{\text{loss}}=\sum (1-\text{CE})\times C$$ (1)

As shown in Fig. 1B, *C*_loss_ increases approximately linearly during the initial stable cycles, whereas deviations from linearity occur as CE begins to degrade. The marked transition points represent the number of stable cycles (*N*) and the corresponding threshold value of *C*_loss_, defined as *C*_loss threshold_. *N* can be correlated with *C*_loss threshold_, average CE (CE_avg_), and *C*, described as

$$N=\frac{C_{\text{loss threshold}}}{C\times (1-{\text{CE}}_{\text{avg}})}$$ (2)

Accordingly, the accelerated degradation of Li metal anodes at a high areal capacity, i.e., decreased *N* at a high *C*, can be attributed to two factors that respectively contribute to the increased denominator and decreased numerator in Eq. 2, as detailed in the following.

One factor is the fast increasing *C*_loss_ with increasing *C*, as indicated by the steeper slope with increasing *C* in Fig. 1B. This corresponds to increased denominator in Eq. 2. As *C*_loss_ is equal to the total amount of inactive Li that consists of Li^+^ compounds in the SEI (SEI Li^+^) and electrically isolated metallic Li wrapped by SEI (dead Li^0^), a high *C* is accompanied by fast accumulation of highly resistive SEI layer, which is one known cause of Li anode degradation (*18*). Such phenomenon is further validated by electrochemical impedance spectroscopy (EIS) in fig. S2, where the cell with a high *C* shows fast increasing SEI resistance (fig. S2).

Another factor is decreasing *C*_loss threshold_ with increasing *C*, as detailed in Fig. 1C. This corresponds to decreased numerator in Eq. 2. This can be ascribed to the increasingly higher proportion of SEI Li^+^ in inactive Li with increasing *C* (*7*). As SEI derives from the decomposition of electrolyte, a high *C* leads to accelerated electrolyte depletion, resulting in fast degradation of Li metal anodes.

Extending the life span of high–areal capacity Li metal anodes, i.e., increasing *N* at a high *C* in Eq. 2, necessitates the increase of *C*_loss threshold_. As *C*_loss threshold_ appears where CE begins to degrade, it represents the onset of accelerated Li anode degradation that can be triggered by high impedance and electrolyte depletion. Therefore, increasing *C*_loss threshold_ requires alleviation of SEI impedance buildup and irreversible electrolyte consumption, both of which can be correlated with the design of Li host structures (*19*, *20*). In this respect, *C*_loss threshold_ can be identified as a collective descriptor that correlates the durability of Li metal anodes with the design rationale of Li host structures.

Replacing the planar Cu substrate with three-dimensional (3D) carbon substrate has known to be effective in improving the life span of Li metal anodes (*13*), as verified in the comparison between Li||3D carbon fabric (CF; fig. S3) cells in Fig. 1D and Li||Cu cells in Fig. 1A. Such improvements correspond to obviously increased *C*_loss threshold_ (Fig. 1, E and F), despite the similar trend of *C*_loss_ in their initial cycles (fig. S5). Meanwhile, we found that the 3D CF substrate renders a much lower SEI resistance at a similar *C*_loss_ (fig. S6). Therefore, 3D CF substrate with interconnected conductive structure can effectively alleviate the detrimental effect of SEI buildup, leading to increased *C*_loss threshold_ and thus improved life span of Li metal anodes.

However, the Li cycling stability on 3D CF substrate at a high areal capacity of 9 mAh cm^−2^ is still unsatisfactory. Scanning electron microscopy (SEM) images in fig. S7 show that part of the plated Li exhibits whisker-like morphology with large surface area, which tends to induce increased electrolyte decomposition. Besides, obvious debris consisting of collapsed SEI layer can be observed after Li stripping (fig. S8), which is a sign of impedance augmentation. These phenomena are caused by irregular Li plating/stripping, which reduce *C*_loss threshold_ and shorten the life span of high–areal capacity Li metal anodes. As a consequence, although the macroscopic 3D matrix can extend the *C*_loss threshold_ to the levels beyond planar electrode, the lack of control over the Li nucleation and growth dynamics will limit the applicability of 3D matrix for realistic anode design.

### HVDG for enduring deep Li cycling

**Fig. 2.**
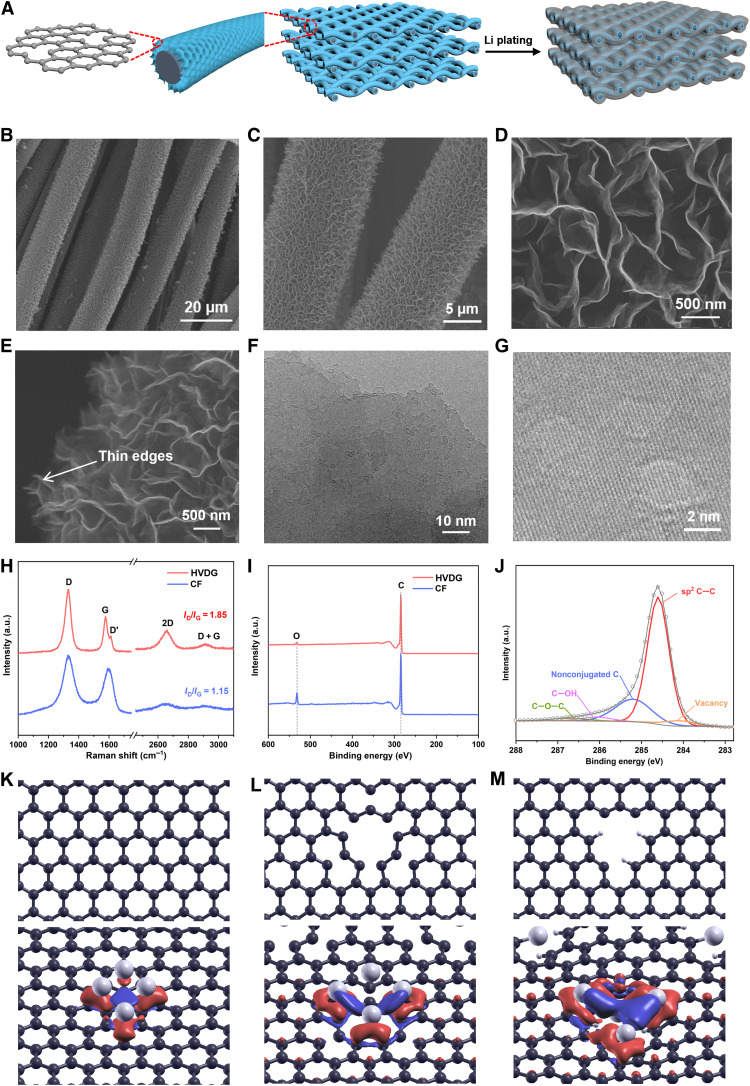
Structure of HVDG. (**A**) Illustration of high–areal capacity Li anode constructed from HVDG. (**B** and **C**) SEM images of the HVDG highlighting the hyperbranched network of vertical graphene arrays. (**D**) SEM image showing the vertical voids surrounded by the interconnected graphene nanosheets. (**E**) SEM image focusing on the edges of vertical graphene nanosheets. (**F**) TEM and (**G**) high-resolution TEM images of HVDG revealing the abundant surface defects on graphene. (**H**) Comparison of Raman spectra of HVDG and CF. a.u., arbitrary units. (**I**) Comparison of XPS spectra of HVDG and CF. (**J**) XPS C1s spectrum of HVDG. (**K** to **M**) DFT calculations showing the geometrical structures of three graphene model surfaces with and without the adsorption of a four-atom Li cluster: (K) perfect graphene, (L) defective graphene with vacancy, and (M) defective graphene with vacancy and hydrogenated carbon. The C, H, and Li atoms are shown in black, white, and silver colors, respectively. The plots of the charge density differences are given in the bottom panels, where the red and blue colors represent electron accumulation and depletion, respectively.

Here, we postulate that an ideal Li host structure that renders a high *C*_loss threshold_ requires essentially macroscopic percolating conductive network and, more importantly, well-engineered atomic-to-microsocpic structures that can induce spatially homogeneous Li plating/stripping with high reversibility. As a proof of concept, HVDG is proposed for enduring deep Li deposition and stripping (Fig. 2A). The highly dispersed atomic defects enable effectively homogenized Li nucleation growth, the low-tortuosity microstructures of the vertical cavity of the graphene arrays are highly beneficial for uniform Li^+^ stripping with minimized inactive Li formation (*7*), and the hyperbranched architecture facilitates charge transfer. These structural features are found highly beneficial for high *C*_loss threshold_ that is responsible for durable deep Li cycling.

HVDG is synthesized through plasma-enhanced chemical vapor deposition (PECVD) using CF as substrate (*21*). The loading density of vertical graphene arrays in the HVDG is ~0.4 mg cm^−2^. The x-ray diffraction (XRD) pattern of HVDG is almost identical to that of the bare CF (fig. S9), with typical diffraction peaks at 26° and 43° that represent the graphite structure. The nitrogen adsorption-desorption isotherms of HVDG and CF are shown in fig. S10, and the specific surface area of HVDG (4.02 m^2^ g^−1^) is slightly higher than that of CF (0.97 m^2^ g^−1^). SEM images in Fig. 2 (B and C) show that the graphene arrays with interconnected morphology are perpendicular to the surface of carbon fibers, with a thickness of about 1.2 μm (fig. S11). The graphene arrays exhibit a self-supported structure featuring continuous network of interlocking graphene sheets. The cavities encircled by adjacent graphene nanosheets have a size of 400 to 600 nm (Fig. 2D). Such vertical arrays of conductive graphene provide sufficient accessible sites for Li nucleation/deposition, effectively reducing the local current density to promote uniform Li deposition (*20*, *22*, *23*). The graphene edge planes are thin and sharp (Fig. 2E), because the graphene nanosheets tend to taper off at their outer edges with a few graphene layers at the base and atomically thin layers formed at the edge (*24*). According to Clar’s aromatic sextet theory, the presence of exposed edges can modulate the original uniform sextet distribution in graphene and a high density of electrons can be localized in regional areas of edge-rich graphene, which is beneficial for low-barrier Li nucleation (*25*, *26*). Figure 2F shows the transmission electron microscopy (TEM) image of the defective graphene, where abundant defects with a size of around 2 nm can be observed. High-resolution TEM image in Fig. 2G indicates that the defects exist on single sheets in the few-layer region of graphene sheets.

Raman spectroscopy and x-ray photoelectron spectroscopy (XPS) were used to further determine the defective structures in the HVDG. Raman spectra of the HVDG and bare CF are compared in Fig. 1H. The G band at 1577 cm^−1^ represents the *E*_2g_ vibrational mode of sp^2^-bonded carbon, and the 2D band at 2670 cm^−1^ is a second-order vibration caused by the scattering of phonons at the zone boundary phonons, which is a prominent feature of graphitic structure (*27*). The D band at 1330 cm^−1^ is a characteristic feature depicting defects and disorder. The ratio of intensities of D band and G band is obviously higher in the HVDG (*I*_D_/*I*_G_ = 1.85) than that in bare CF (*I*_D_/*I*_G_ = 1.15), indicating higher level of defects in the HVDG. The average distance between defects (*L*_D_) in the HVDG is calculated to be 7.4 nm (*I*_D_/*I*_G_ = 102/*L*_D_^2^) (*28*), lower than that in the bare CF (9.4 nm). Besides, D′ band appears at around 1615 cm^−1^ as a shoulder of G band in the Raman spectra of the HVDG, which is another indicator of structural disorder in graphitic materials (*29*). XPS spectra in Fig. 1I show that the content of oxygen in the HVDG is negligible, indicating that the defective structures in the HVDG are mainly originated from intrinsic carbon defects rather than oxygen-containing functional groups. The local bonding environment of carbon atoms in the HVDG is further analyzed in the C1s spectrum in Fig. 1J. Two peaks stemming from the defects are found next to the prominent peak at 284.6 eV for sp^2^ C─C bond. The intensity ratio of these defects to sp^2^-hybridized carbon is 0.35 (calculated on the basis of the integral areas of the peaks), indicative of a high defect population. The peak centred at 284.1 eV represents the carbon vacancies at the lattice sites, and the peak at 285.2 eV can be assigned to nonconjugated carbon that mostly include hydrogenated carbon (*30*, *31*). Vacancies in graphene have been proved able to induce the formation of Li-intercalated states with a high lithium-to-carbon ratio such as Li_3_C_8_ (*32*), compared to the common LiC_6_. It has been well established that Li metal nucleation/plating is favored at sites that display large charge concentration (*33*). Therefore, these Li-intercalated states with high concentration of Li ions can promote uniform Li nucleation/deposition. For the hydrogenated carbon, it has been experimentally verified by scanning tunneling microscopy that high-concentration electrons can be localized in some specific parts of hydrogen-terminated graphene nanosheets (*26*, *34*). Such strong regional electronegativity is beneficial to attracting Li ions for uniform Li nucleation with low barriers. We further implemented density functional theory (DFT) calculations to investigate the interaction between Li and the defective graphene (fig. S12). It is found that such defective graphene structure with both vacancy and hydrogenated carbon (G_defc+H_) renders strong binding interactions with Li, compared to perfect graphene (G_perf_) and defective graphene with vacancy only (G_defc_) (Fig. 1, K to M, and fig. S12). The calculated adsorption energy of the Li cluster on the G_defc+H_ was also found much higher than those on the G_perf_ and G_defc_ (fig. S12B), indicating thermodynamically favorable Li nucleation on the HVDG.

**Fig. 3.**
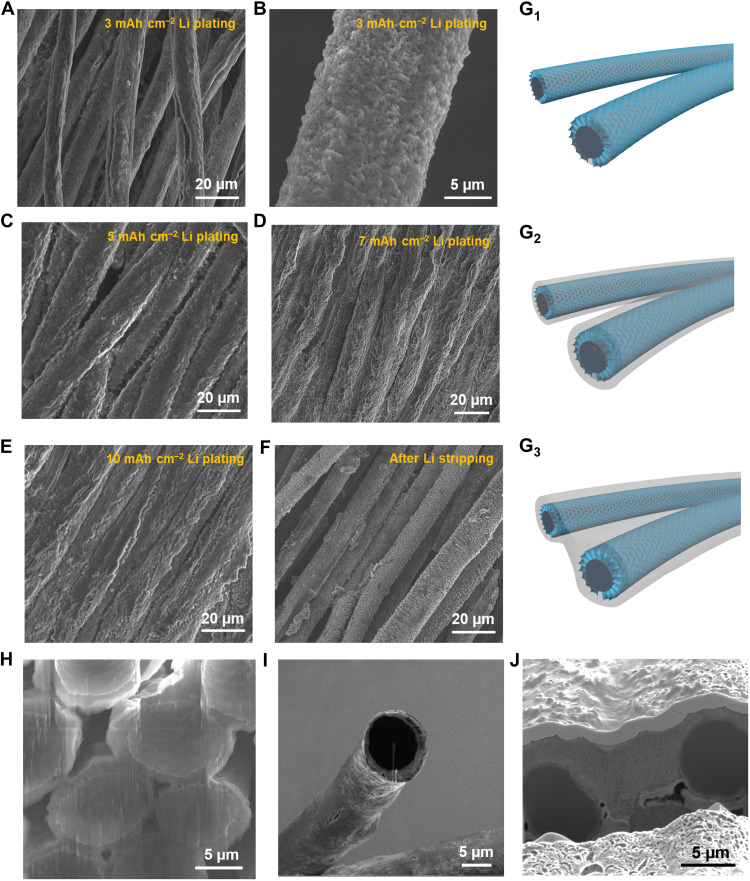
Morphological evolution of Li plating and stripping on HVDG. (**A** to **F**) SEM images showing the morphological evolution of Li-HVDG with Li plating capacities of (A and B) 3 mAh cm^−2^, (C) 5 mAh cm^−2^, (D) 7 mAh cm^−2^, (E) 10 mAh cm^−2^, and (F) after Li stripping. The current density for Li plating/stripping is 1 mA cm^−2^. (**G**) Schematic diagrams of Li plating behavior on HVDG at (G_1_) low, (G_2_) moderate, and (G_3_) high Li plating capacities. (**H** to **J**) Cross-sectional SEM image of Li-HVDG with Li plating capacities of (H) 3 mAh cm^−2^, (I) 5 mAh cm^−2^, and (J) 10 mAh cm^−2^ (the layer on the upper surface is the Pt protection layer).

SEM images in Fig. 3 (A to E) show the morphological evolution of Li-HVDG with increasing Li plating capacities up to 10 mAh cm^−2^. The corresponding cross-sectional SEM images are shown in Fig. 3 (H to J). During the initial Li plating stage, the deposited Li preferentially fills the vertical cavities among the graphene arrays (Fig. 3, A, B, and H). As validated by the simulation results of COMSOL Multiphysics (fig. S13), the Li ion flux density within the cavities is more concentrated than that on the upper surface, resulting in preferential nucleation of Li inside the vertical cavities. It is known that the formation of Li nuclei on Li metal is thermodynamically favorable with eliminated nucleation barrier, owing to the identical crystal structures of pure Li metal (*35*). Therefore, the initially deposited Li with spatially homogeneous structure can act as seeds to guide uniform formation of new Li nuclei during the subsequent Li plating process, resulting in a uniform Li coating on the fiber (Fig. 3, C and I). As the Li plating capacity further increases, the Li deposits gradually fill up the residual space within the HVDG (Fig. 3, D, E, and J), resulting in a high–areal capacity Li anode with spatial homogeneity. Illustrations of Li-HVDG at different stages of Li plating are shown in Fig. 3 (G_1_ to G_3_). The uniform microstructures of Li deposits are highly beneficial for the formation of spatially homogeneous SEI (*7*), which contributes to uniform Li dissolution during the Li stripping process. After stripping of plated Li (10 mAh cm^−2^), the hyperbranched structure of HVDG can be well preserved, and much less agglomerates of dead Li/SEI debris are observed (Fig. 3F), compared to that of the bare CF (fig. S8), indicating highly reversible Li plating/stripping at a high areal capacity in HVDG. SEM images of Li-HVDG after Li stripping at higher resolutions in fig. S14 reveal that the walls of the vertical graphene arrays become coarse compared to their pristine morphology (Fig. 2, D and E), which can be ascribed to the formation of SEI on the surface of graphene.

**Fig. 4.**
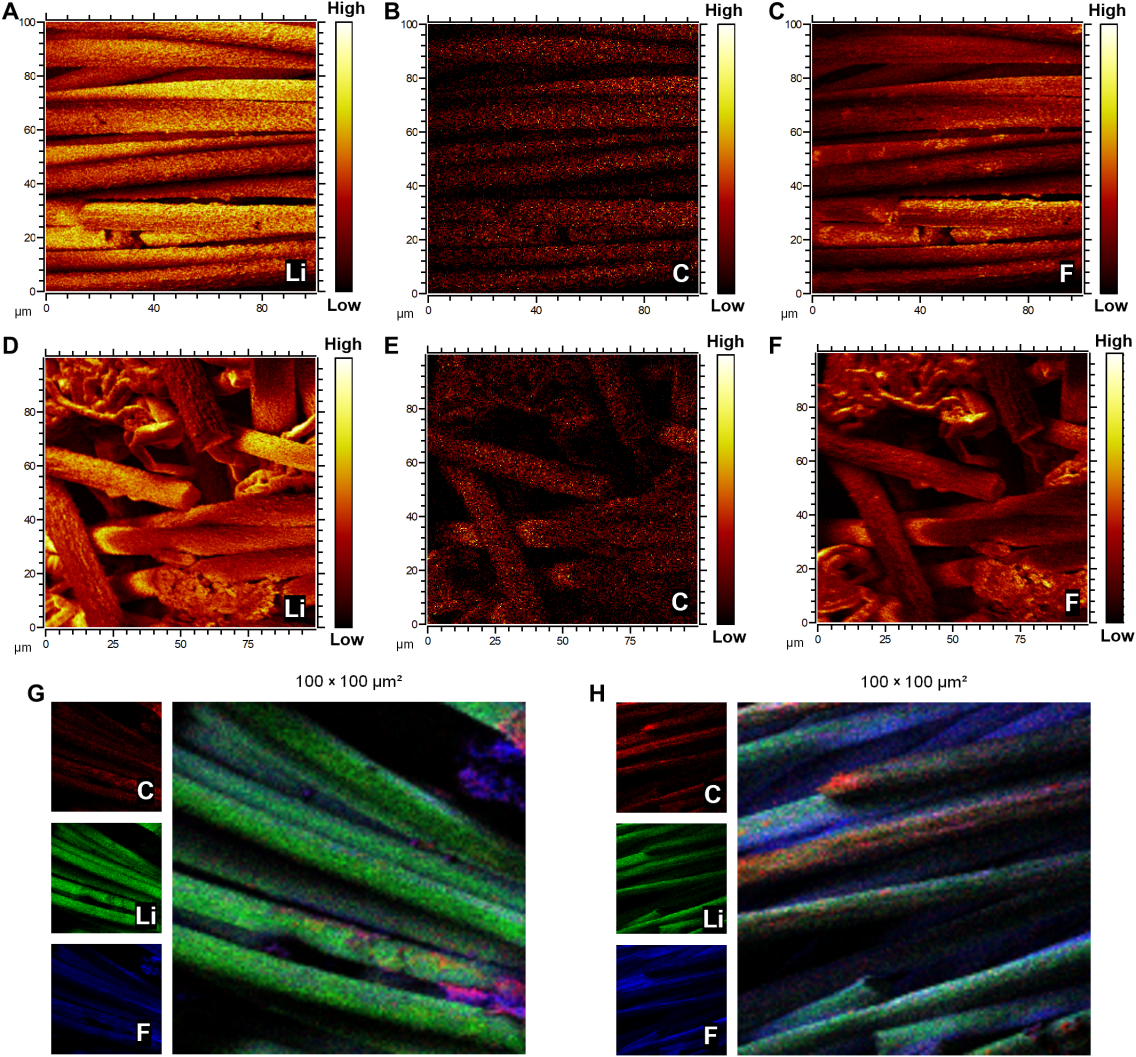
ToF-SIMS characterizations of Li plating and stripping. ToF-SIMS secondary ion mapping of (**A** to **C**) Li-HVDG and (**D** to **F**) Li-CF with Li plating of 5 mAh cm^−2^, and (**G**) HVDG and (**H**) CF after Li stripping.

The distributions of Li and SEI components during the Li plating/stripping process are further scrutinized through secondary ion imaging maps obtained from time-of-flight secondary ion mass spectrometry (ToF-SIMS). Figure 4 (A to C) shows the ToF-SIMS maps of Li, C, and fluorine (F) in Li-HVDG with plated Li (5 mAh cm^−2^), where the existence of F arises from electrolyte decomposition and can be regarded as a distinctive signal for SEI. The signals of Li elements are highly consistent with those of C and F elements, indicating uniform SEI distribution. For comparison, the corresponding ToF-SIMS maps of Li-CF with the plated Li (5 mAh cm^−2^) are given in Fig. 4 (D to F). As shown in Fig. 4D, part of the Li deposits present as dendrite-like agglomerates, in accordance with the SEM images in fig. S7. The nonuniform SEI distribution associated with irregular Li agglomerates is reflected in the mapping of F in Fig. 4F. The ToF-SIMS mappings of the HVDG and CF after Li stripping are shown in Fig. 4 (G and H), respectively. The detected signal of Li after Li stripping mainly comes from the SEI Li. Good alignment of C, Li, and F elements in Fig. 4G indicates homogeneous SEI covered on the surface of the HVDG, while the obvious misalignment of elemental mappings in Fig. 4H indicates the interfacial heterogeneity of CF. The misalignment of elemental mappings in the right corner of Fig. 4G should be originated from the SEI debris after Li stripping, which can also be observed in the SEM image in Fig. 3F. Such comparisons reveal that the HVDG with highly dispersive defects and vertical cavities can facilitate uniform Li deposition and SEI formation, contributing to high Li plating/stripping efficiency.

**Fig. 5.**
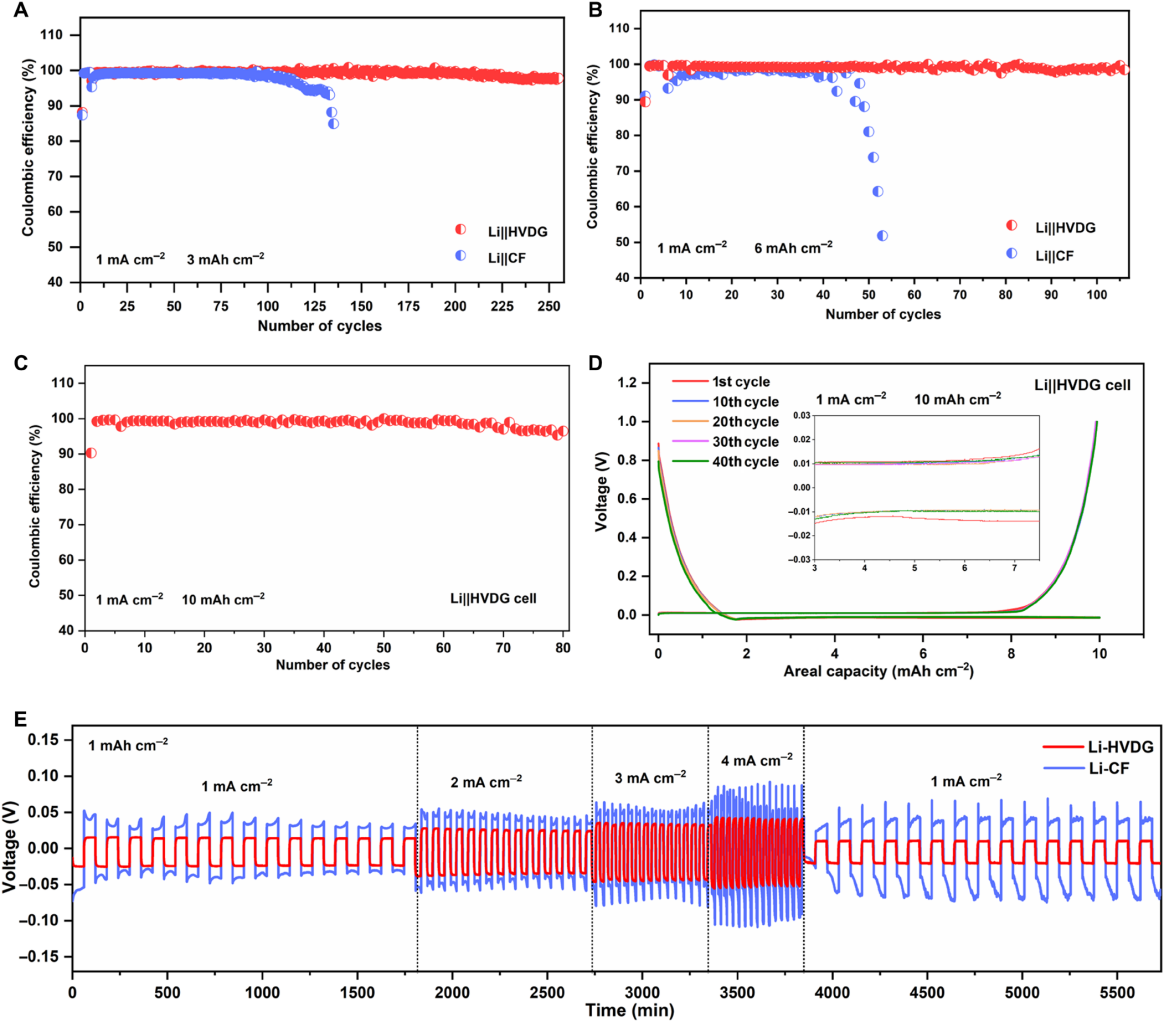
Electrochemical characterizations of the Li-HVDG anodes. The comparisons of CEs of Li||HVDG cell and Li||CF cell with a Li plating capacity of (**A**) 3 mAh cm^−2^ and (**B**) 6 mAh cm^−2^ at 1 mA cm^−2^. The CEs of the initial five formation cycles between 0 and 1 V are also included. (**C**) CE and (**D**) voltage profiles of Li||HVDG cell with an areal capacity of 10 mAh cm^−2^ at 1 mA cm^−2^. The CEs of the initial five formation cycles between 0 and 1 V are also included. (**E**) Voltage profiles of Li-HVDG and Li-CF symmetric cells at current densities from 1 to 4 mA cm^−2^ with a Li stripping/plating capacity of 1 mAh cm^−2^.

To further elucidate the effect of HVDG on long-term deep Li cycling, we show comparisons of CEs of the Li||HVDG cell and Li||CF cell in Fig. 5 (A and B). At an areal capacity of 3 mAh cm^−2^, the Li||HVDG cell delivers stabilized CE for 250 cycles, while the Li||CF cell experiences obvious decay of CE after 100 cycles (Fig. 5A). When increasing the areal capacity to 6 mAh cm^−2^, the CE of the Li||HVDG cell can be maintained at ~99% over 100 cycles (Fig. 5B). In contrast, the CE of the Li||CF cell quickly drops to below 50% after only 48 cycles. At a high areal capacity of 10 mAh cm^−2^, the Li||HVDG cell maintains stabilized CE for 80 cycles (Fig. 5C), which corresponds to a high *C*_loss threshold_ of 9.67 mAh cm^−2^. EIS profiles of the Li||HVDG cell after 1st and 80th cycles are given in fig. S15. With such a high *C*_loss threshold_, the cell impedance exhibits no sudden increase, indicating homogeneously distributed SEI through the hyperbranched conductive network. The corresponding charge/discharge profiles in Fig. 5D show negligible changes in voltage plateaus upon cycling, which further indicates good reversibility and stability of the Li-HVDG anode. In contrast, the Li||CF cell undergoes increased charge-discharge voltage polarization upon cycling at 10 mAh cm^−2^ (fig. S16). Figure S17 shows the CE test of the Li||HVDG cell at a higher current density of 3 mA cm^−2^, where long-term stability can be maintained for 345 cycles. Figure 5E shows the voltage profiles of Li-HVDG and Li-CF symmetric cells, where the cell with Li-HVDG electrodes exhibits smaller voltage hysteresis, indicating improved kinetics for Li plating/stripping (*36*). A similar trend was observed when increasing the cycling capacity to 3 mAh cm^−2^ (fig. S18). To further elucidate the advantage of the trans-scale design of HVDG, we also fabricated vertical defective graphene arrays grown on planar Cu (VDG-Cu) for comparison (fig. S19A), which features atomic-to-microscopic design. Figure S19B shows the CE of the Li||VDG-Cu cell under areal capacities of 3 mAh cm^−2^ at 1 mA cm^−2^, where stable cycles can be maintained for 40 cycles. Such electrochemical performance is better than that with the bare Cu substrate (Fig. 1A) but cannot outperform both the 3D CF substrate and HVDG, which highlight the significance of the trans-scale design concept.

To verify the potential of the Li-HVDG anode in practical applications, we assembled full cells using Li-HVDG as the anode and commercial LiFePO_4_ (LFP) as the cathode. The areal capacity of the cathode is ≥4 mAh cm^−2^ to meet the practical requirements for LMBs (*2*, *15*). The areal capacity of the anode is adjusted on the basis of different N/P ratios. Li-Cu||LFP cells were assembled for comparison. The electrolyte amount added in these full cells varies on the basis of different electrolyte-to-capacity (E/C) ratios.

**Fig. 6.**
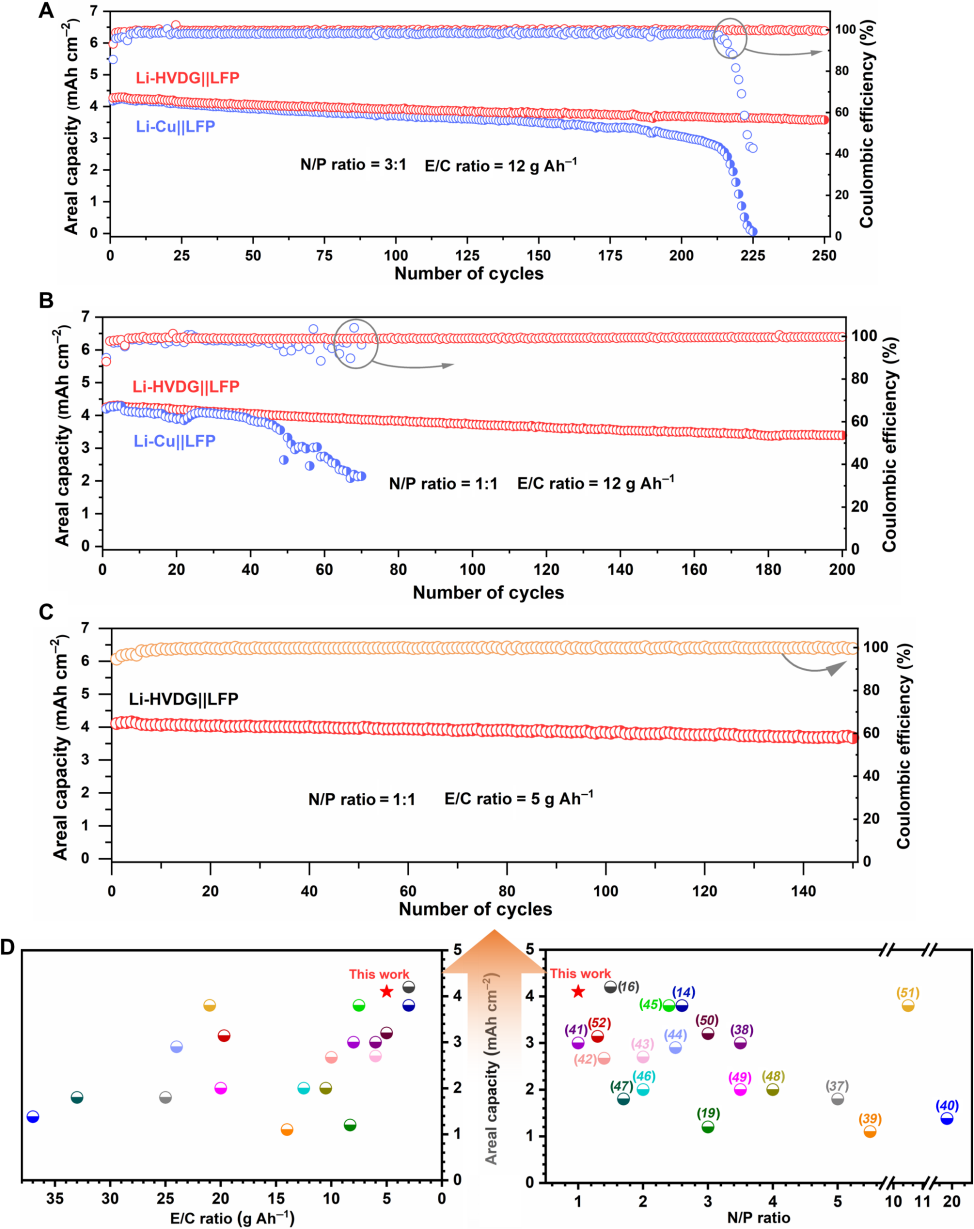
Electrochemical performance of Li metal full cells. (**A** to **B**) Comparisons of cycling performance of Li-HVDG||LFP cell and Li-Cu||LFP cell at 0.2 mA cm^−2^ with (A) an N/P ratio of 3:1 and an E/C ratio of 12 g Ah^−1^ and with (B) an N/P ratio of 1:1 and an E/C ratio of 12 g Ah^−1^. (**C**) Cycling performance of Li-HVDG||LFP cell at 0.15 mA cm^−2^ with an N/P ratio of 1:1 and an E/C ratio of 5 g Ah^−1^. (**D**) Comparisons of areal capacity versus N/P ratio and E/C ratio between the Li-HVDG||LFP cell in this work and recently reported Li metal full cells (*14*, *16*, *19*, *37*–*52*).

Figure 6A shows the comparisons of cycling performance of the Li-HVDG||LFP and Li-Cu||LFP cells at 0.2 mA cm^−2^ with an N/P ratio of 3:1 and an E/C ratio of 12 g Ah^−1^. The Li-HVDG||LFP cell exhibits long-term cycling stability over 250 cycles with an average CE of 99.7% (fig. S20A). The Li-Cu||LFP cell shows comparable stable cycling during the initial cycles, followed by gradual capacity decay from the 150th cycle and a sudden capacity drop from the 200th cycles. The initial stable cycles of the Li-Cu||LFP cell can be ascribed to the large excess of Li anode and flooded electrolyte supply, which can compensate for the irreversible Li loss and electrolyte consumption. Once the excess Li or the electrolyte is depleted, the cell can be suddenly terminated. When the cells were tested under a restricted N/P ratio of 1:1, the Li-Cu||LFP cell can survive for only 40 cycles (Fig. 6B), indicating severe irreversible loss of electroactive Li when plated/stripped on the Cu substrate. In contrast, the Li-HVDG||LFP cell demonstrates stable cycles over 200 cycles with a capacity retention rate of 80% and an average CE of 99.2% (fig. S20B). Rate capabilities of the Li-HVDG||LFP cell are shown in fig. S21A. When the current density increases from 0.3 to 1.5 mA cm^−2^, the cell delivers 80% of the capacity at 0.3 mA cm^−2^. Figure S21B displays the corresponding charge/discharge profiles at different current densities. The charge/discharge plateaus remain flat and stable even at high current densities, suggesting favorable charge transfer kinetics.

To demonstrate a prototype high-energy Li metal cell with high areal capacity, low N/P ratio, and lean electrolyte supply, we further reduced the E/C ratio to 5 g Ah^−1^. As shown in Fig. 6E, the cell with an N/P ratio of 1:1 and an E/C ratio of 5 g Ah^−1^ maintains good stability for 150 cycles and an average CE of 99.7% (fig. S20C). The corresponding charge/discharge profiles upon cycling are given in fig. S22, where the charge/discharge plateaus are well retained during cycling without obvious increase in overpotential. When comparing with the recently reported Li metal full cells (*14*, *16*, *19*, *37*–*52*), the Li-HVDG||LFP cell in this work is more appropriately positioned at a high areal capacity, low N/P ratio, and low E/C ratio (Fig. 6D and table S1), highly desirable for achieving a high practical energy density. Such promising performance obtained in alignment with realistic cell conditions unambiguously demonstrates the effectiveness of the Li-HVDG electrode that enables enduring deep cycling of Li metal.

## DISCUSSION

Improving cell energy density while achieving enduring cycling stability has been a grand challenge for Li metal batteries, owing to the accelerated degradation of high–areal capacity Li metal anodes upon stripping and plating. This work aims to establish a design principle for Li host structures that promise deep cycling of Li metal with high reversibility and durability. We hope to inspire more research interest in explorations of the correlation between Li electrochemical cycling behavior and material structures and properties as well as the corresponding underlying mechanisms. It is worth noting that, besides Li anode structure design, optimization of electrolyte compositions is another important route for stabilization of Li metal anodes. It is highly desirable to develop electrolytes with both wide voltage window and good compatibility with Li metal, so that the proposed trans-scale design concept for Li metal anodes can be further incorporated in Li metal cells with more aggressive high-voltage cathodes such as lithium nickel cobalt manganese oxides (NCM) and lithium nickel cobalt aluminium oxides (NCA), to further boost the specific energy of Li metal batteries with potential use in emerging high-energy applications like electric vehicles. The interplays among cathode chemistry, electrolyte engineering, SEI/dead Li formation, and material design principles can be further investigated in the future studies. In addition HVDG can possibly be applied in many other areas like biosensors, strain sensors, piezoresistive flow sensors, and water filtration and disinfection.

In summary, we report the design of HVDG for enduring deep Li cycling at practical-level high areal capacities. The trans-scale atomic-to-macroscopic electrode design rationale is validated by quantifying the degradation dynamics of Li metal anodes and correlated with a descriptor that dictates the durability of high–areal capacity Li metal anodes. The highly dispersed defects and vertical geometry of graphene arrays, combined with the hyperbranched architecture, lead to effective spatial control of Li plating/stripping with high reversibility and durability. High-energy LMB cells are prototyped under realistic conditions with a high areal capacity ≥4 mAh cm^−2^, a low N/P ratio of 1:1, and a low E/C ratio of 5 g Ah^−1^. This work shed light on a promising move toward practical Li metal batteries.

## MATERIALS AND METHODS

### Preparation of the HVDG

HVDG is synthesized via a PECVD process using a commercial CF (CeTech, 9 mg cm^−2^, 100 μm) as substrate. CF was loaded into a plasma chamber. When the base pressure inside the plasma chamber reached 0.02 Pa, Ar gas was introduced at a flow rate of 10 standard cubic centimeters per minute (sccm) until the chamber pressure was raised to 1.5 Pa. Plasma was then ignited by a radio-frequency power source (13.56 MHz) at 1000 W. The Ar plasma pretreatment can clean the surface of CF and improve the adhesion between vertical graphene arrays and CF. After 10 min, CH_4_ (20 sccm) and H_2_ (10 sccm) were introduced into the chamber as the precursor gases for the growth of vertical graphene arrays. The chamber pressure was kept at 1.9 Pa, and the growth temperature was maintained at ca. 400°C by the plasma. After 10 min of growth, the HVDG was obtained. The weight of the obtained HVDG remains almost unchanged compared to the pristine CF because of plasma-induced itching of the substrate during the growth of graphene.

### Characterizations

The morphology and structure of the samples were characterized using a scanning electron microscope (FEI Nova NanoSEM 450, 15 kV) and an aberration-corrected transmission electron microscope (JEOL JEM-ARM300F2, 60 kV). The cross-sectional SEM images of Li-HVDG were collected with a Helios focused ion beam SEM instrument (FEI Helios G4 PFIB UXe DualBeam system) with 2.5-μA Xe plasma FIB ion beam. The cross-sectional milling process was started by coating a Pt layer on the Li-HVDG and performed step by step at a voltage of 30 kV. The final polishing process was carried out at a voltage of 3 kV. XRD was conducted on a D8 (Bruker) Thin-Film XRD with Cu Kα radiation (λ = 1.54056 Å). Specific surface area was measured using a Micromeritics TriStar 3030 instrument using the nitrogen physisorption technique at −195.8°C. Before analysis, the samples were degassed at 150°C for 3 hours under vacuum. XPS analysis was performed using a Thermo ESCALAB250i instrument with Al Kα radiation (15 kV, 150 W). ToF-SIMS (ION-TOF TOFSIMS 5) was performed using a bismuth cluster analysis beam (30 keV). Raman measurements were performed using a Renishaw inVia 2 Raman Microscope with an excitation wavelength of 633 nm.

### Preparation of the cathode

The cathode slurry was prepared by mixing the LFP powder (MTI corporation) with polyvinylidene fluoride (MTI) and carbon black (MTI) in a ratio of 96:2:2 using *N*-methyl-2-pyrrolidone (Sigma-Aldrich) as the solvent. To obtain high-mass loading cathode, a carbon fiber foam prepared from carbonization of natural cotton (*53*) was used as the cathode current collector (areal density, 4 mg cm^−2^). The carbon fiber foam was immersed into the cathode slurry for 10 s, and then, it was removed from the slurry and placed in a vacuum oven at 100°C overnight to obtain the LFP cathode. The dried LFP cathode was punched into disks with a diameter of 12 mm for cell assembly. The areal mass loading was controlled to 28 to 32 mg cm^−2^ to reach an areal capacity >4 mAh cm^−2^.

### Electrochemical measurements

All cells were assembled using CR2032-type coin cells (MTI) in an Ar-filled glove box [O_2_ < 0.1 parts per million (ppm), H_2_O < 0.1 ppm]. The electrolyte was prepared by dissolving Li bistrifluoromethanesulphonylimide (99%, Sigma-Aldrich, 1 M) and lithium nitrate (LiNO_3_, 99.9%, Sigma-Aldrich, 0.2 M) in 1,2-dimethoxyethane (99.5%, Sigma-Aldrich) and 1,3-dioxolane (99.5%, Sigma-Aldrich) (1:1 ratio, by volume). Celgard 2400 was used as the separator. Cycling and rate performance tests were carried out using a NEWARE galvanostatic charge-discharge instrument. EIS measurements were conducted using a Biologic VSP potentiostat with a frequency range from 100 kHz to 10 mHz.

For Li||Cu cells, Cu foil (diameter of 12 mm) was used as the working electrode for Li metal plating, and the Li foil chip (diameter of 15.6 mm and thickness of 250 μm, from MTI) was used as the counter electrode. The electrolyte amount added in the cell was 50 μl. A given amount of Li metal was plated onto the Cu substrate, followed by stripping Li metal from the Cu substrate to a cutoff voltage of 1 V. The current density was 1 mA cm^−2^. CE is calculated on the basis of the ratio between the amount of Li stripped from the Cu substrate and that deposited on the Cu substrate. Average CE for a given number of cycles (*n*) is calculated by ${\text{CE}}_{\text{avg}}=\frac{1}{n}\sum \text{CE}$.

Li||3D CF and Li||HVDG cells were assembled under the same condition as Li||Cu cells, using CF (diameter of 12 mm) and HVDG (diameter of 12 mm) as the working electrodes, respectively. These cells were first cycled between 0 and 1 V for 5 cycles (formation cycles), following by the same cycling protocols as Li||Cu cells. In particular, the Li||3D CF cells in Fig. 1 were cycled with a charge cutoff voltage of 0.03 V (fig. S4). Such Li cycling protocol could minimize the effect of capacity contribution from the lithiation of CF substrate, resulting in a fair comparison between Li||Cu and Li||3D CF cells in Fig. 1.

For symmetric Li-HVDG and Li-CF cells, Li (5 mAh cm^−2^) was first preplated onto the HVDG and CF using the Li||HVDG and Li||CF cell configurations. These cells were then disassembled to obtain Li-HVDG and Li-CF electrodes. Symmetric Li-HVDG and Li-CF cells were assembled using two identical electrodes. The electrolyte amount added in the symmetric cells was 50 μl.

For full cells, Li-HVDG and Li-Cu anodes were first prepared by plating a given amount of Li onto HVDG (diameter of 12 mm) and Cu (diameter of 12 mm) using the Li||HVDG and Li||Cu cell configurations. Specifically, Li-HVDG and Li-Cu anodes with plated Li (12 mAh cm^−2^) were used to assemble the full cells with an N/P ratio of 3:1, and those anodes with plated Li (4 mAh cm^−2^) were used to assemble the full cells with an N/P ratio of 1:1. The areal capacity of the Li-HVDG anode includes the lithiation capacity of the HVDG (~1.5 mAh cm^−2^). For the full cells with an E/C ratio of 12 g Ah^−1^ (55 μl), the LFP cathode (28 mg cm^−2^) was used. For the full cells with an E/C ratio of 5 g Ah^−1^ (23 μl), the LFP cathode (32 mg cm^−2^) was used. The full cells were tested in a voltage range of 2.5 to 3.8 V.

### COMSOL simulations

The simulation was conducted by the COMSOL Multiphysics software based on Finite Element Method to investigate the Li ion flux distribution near the HVDG electrode. “The Tertiary Current Distribution, Nernst-Planck” module was used. Simulation models of the electrolyte and electrode domains are given in fig. S13A. The top boundary of electrolyte domain is set as anode with the potential of 0.02 V, and the bottom boundary with HVDG is set as cathode with the potential of 0 V. The initial Li^+^ concentration in the electrolyte is 1 M. The diffusion coefficient is set as 4 × 10^−6^ cm^2^ s^−1^. The exchange current density is set as 0.376 A m^−2^. Under the modeled conditions, the free convection between cathode and anode can be neglected as the variations are sufficiently small.

### DFT calculations

DFT calculations were performed using the projector augmented wave method, as implemented in Quantum Espresso. Exchange-correlation energies were obtained using the revised Vydrov–Van Voorhis (rVV10) functional. Kohn-Sham orbitals were expanded in a plane wave basis set with a cutoff energy of 49 and 511 rydberg (Ry) for the charge density cutoff. The Brillouin zone integration was performed by the Methfessel-Paxton special point technique, with a smearing parameter of 0.02 Ry and *k*-point meshes of 3 × 3 × 1. Effective screening medium and fictitious charge particle as the computational approaches were taken to perform constant potential calculations. We use vacuum-slab-metal as the boundary condition for electrochemical systems. The perfect graphene was modeled by a periodically extended system in (5√3 × 5√3) size, which has 50 carbon atoms in total, and was separated by a vacuum region about 18 Å. The defective graphene with vacancy was created by removing four C atoms away from the central of the extended model, and the defective graphene with vacancy and hydrogenated carbon was modeled by attaching three H atoms into the periphery of the resulted multivacancy cavity. The three models, namely, the perfect graphene, defective graphene with vacancy, and defective graphene with vacancy and hydrogenated carbon, with and without deposition of a four-atom Li cluster, were considered for comparative studies. Structural optimizations were performed at a series of different potentials ranging from the cathodic to anodic ones with respect to the potentials of zero charge (PZC). Structure optimizations were performed with the whole slab relaxed until the Hellmann-Feynman forces were lower than 0.001 Ry per atomic unit. A calculation was considered converged when the energy change per atom was less than 10^−5^ Ry and the mean displacement less than 0.001 Å. Structural models and electron density were represented with XCrySDen. Potential-dependent adsorption energy of the Li cluster was calculated by the following relation: *E*_ads_(*P*) = *E*_Li@{I, II,III}_(*P*) − *E*_{I,II,III}_(PZC) − *E*_Li_(PZC).
