# Supplementary material for: Rationalized design of hyperbranched trans-scale graphene arrays for enduring high-energy lithium metal batteries
Source: Sci Adv. 2022 Aug 24;8(34):eadc9961. doi: 10.1126/sciadv.adc9961 (PMC9401611; doi:10.1126/sciadv.adc9961)
Supplement: Supplementary file 1 — Figs. S1 to S22 Table S1 [file sciadv.adc9961_sm.pdf]

Science Advances
AAAS

# Supplementary Materials for

## Rationalized design of hyperbranched trans-scale graphene arrays for enduring high-energy lithium metal batteries

Ruopian Fang *et al.*

Corresponding author: Da-Wei Wang, da-wei.wang@unsw.edu.au

*Sci. Adv.* **8**, eadc9961 (2022)
DOI: 10.1126/sciadv.adc9961

**This PDF file includes:**

Figs. S1 to S22
Table S1

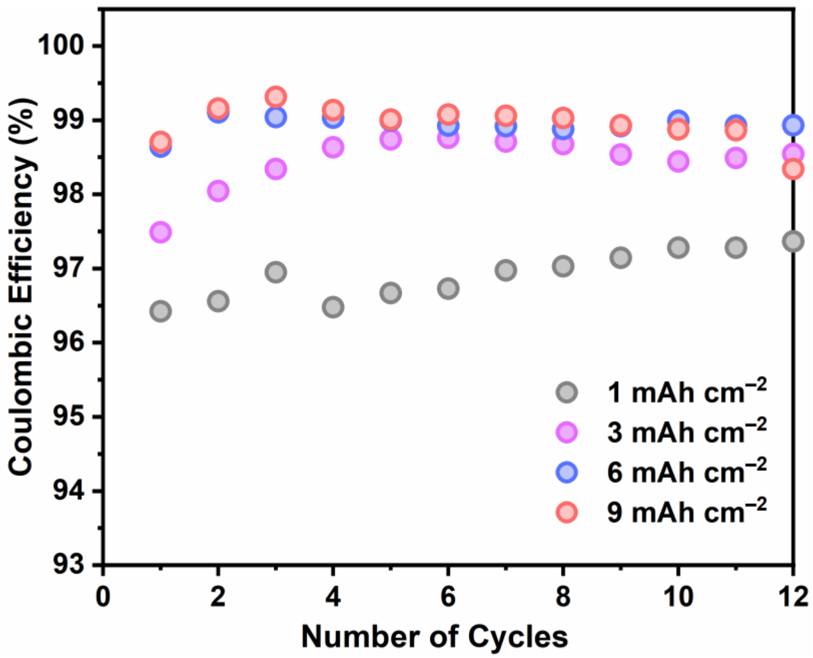


**Fig. S1. Coulombic efficiency of Li||Cu cells in the initial 12 cycles under different areal capacities.**

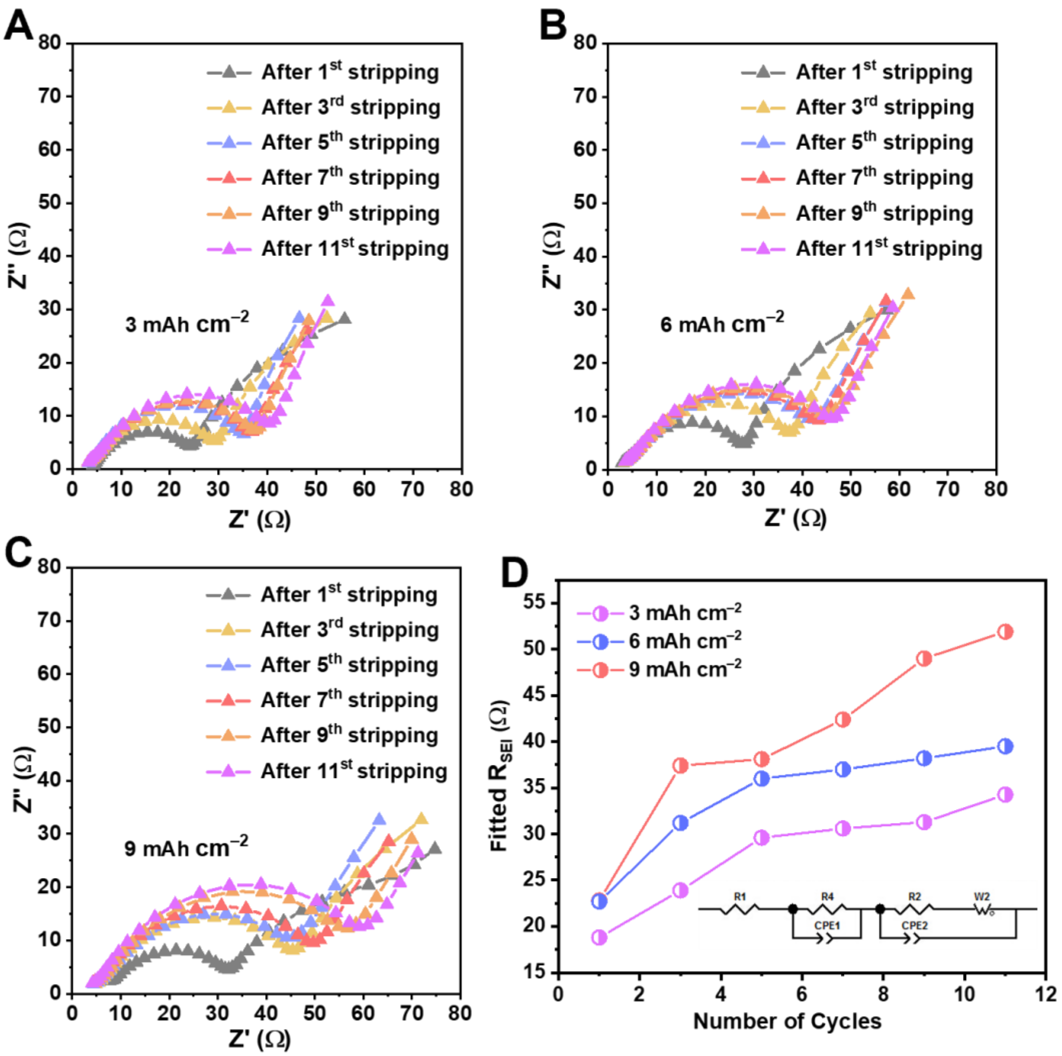


**Fig. S2. Electrochemical impedance spectra (EIS) of Li||Cu cells. (A)** With an areal capacity of 3 mAh $cm^{-2}$. **(B)** With an areal capacity of 6 mAh $cm^{-2}$. **(C)** With an areal capacity of 9 mAh $cm^{-2}$. **(D)** Fitted SEI film resistance ($R_{SEI}$, *i.e.*, $R_4$ in the equivalent circuit in the inset) as a function of number of cycles at different areal capacities.

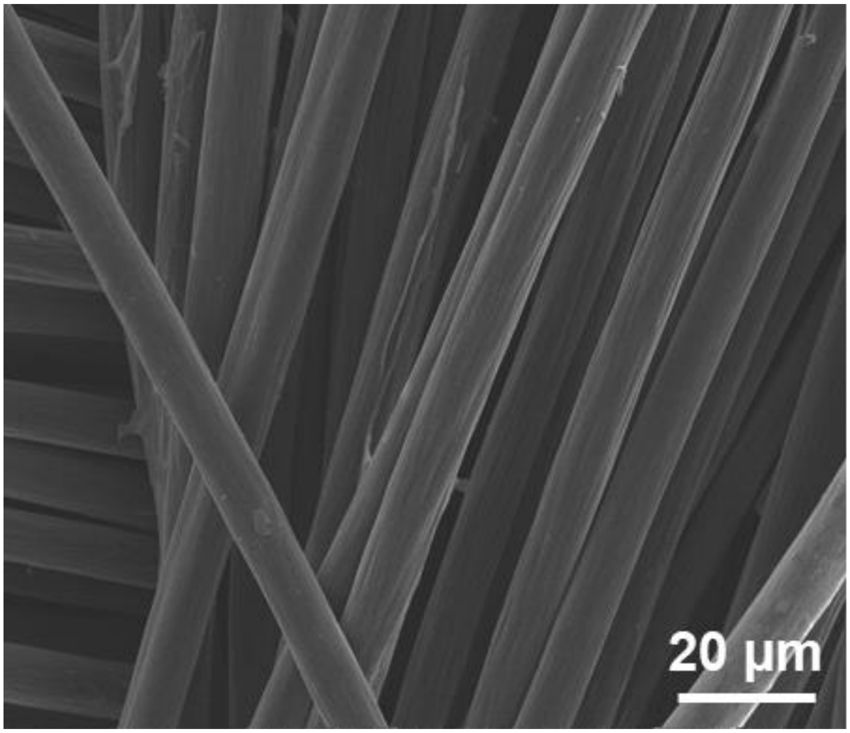


**Fig. S3. SEM image of 3D carbon fibric.**

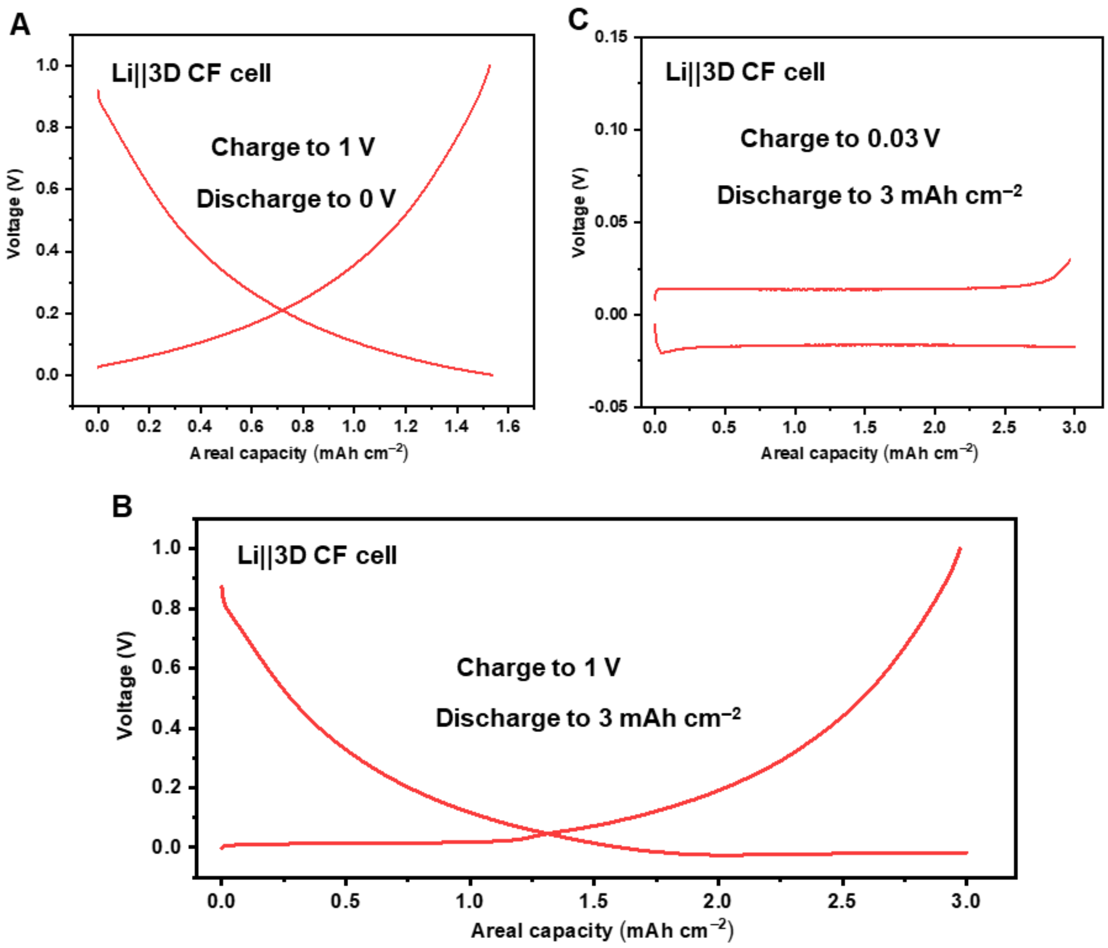


**Fig. S4. Charge/discharge profiles of Li||3D CF cells under different testing conditions**. **(A)** with a voltage window of 0-1V, **(B)** with a Li plating capacity of 3 mAh cm$^{-2}$ and charge cut-off voltage of 1 V, **(C)** with a Li plating capacity of 3 mAh cm$^{-2}$ and charge cut-off voltage of 0.03 V. In order to show a fair comparison between Li||Cu and Li||3D CF cells, the cycling protocol in Fig. S3C was used for Li||3D CF cells measured in Fig. 1, to minimize the effect of the capacity contribution from the lithiation of CF substrate (~ 1.5 mAh cm$^{-2}$).

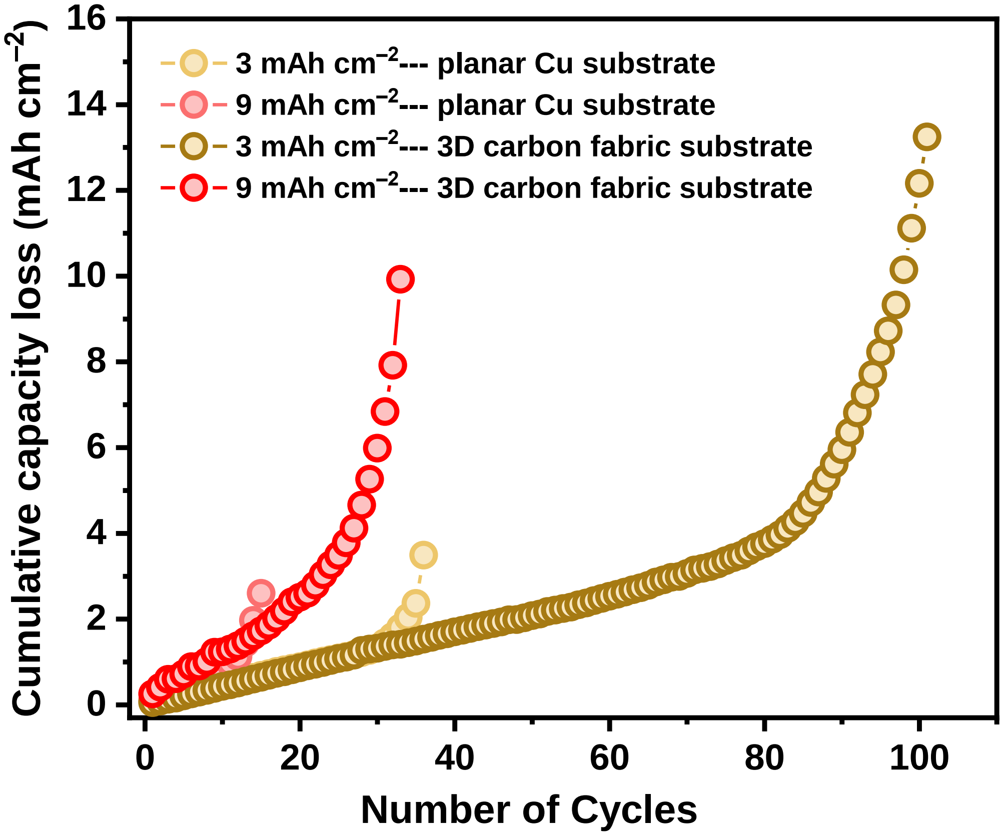


**Fig. S5. Comparison of Cumulative capacity loss as a function of number of cycles in Li||Cu cells and Li||3D CF cells.**

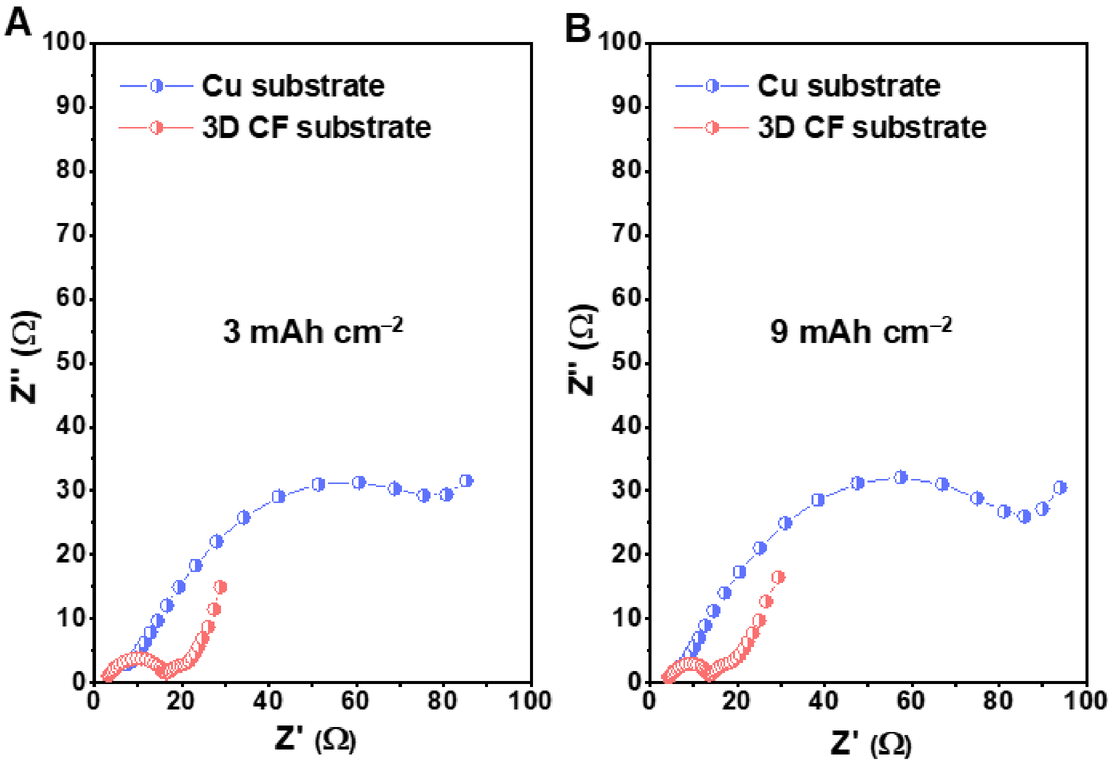


**Fig. S6. Comparisons of EIS profiles of Li||Cu cells and Li||3D CF cells at a similar $C_{loss}$ . (A)** With an areal capacity of 3 mAh cm$^{-2}$ (30$^{th}$ cycle). **(B)** With an areal capacity of 9 mAh cm$^{-2}$ (12$^{nd}$ cycle).

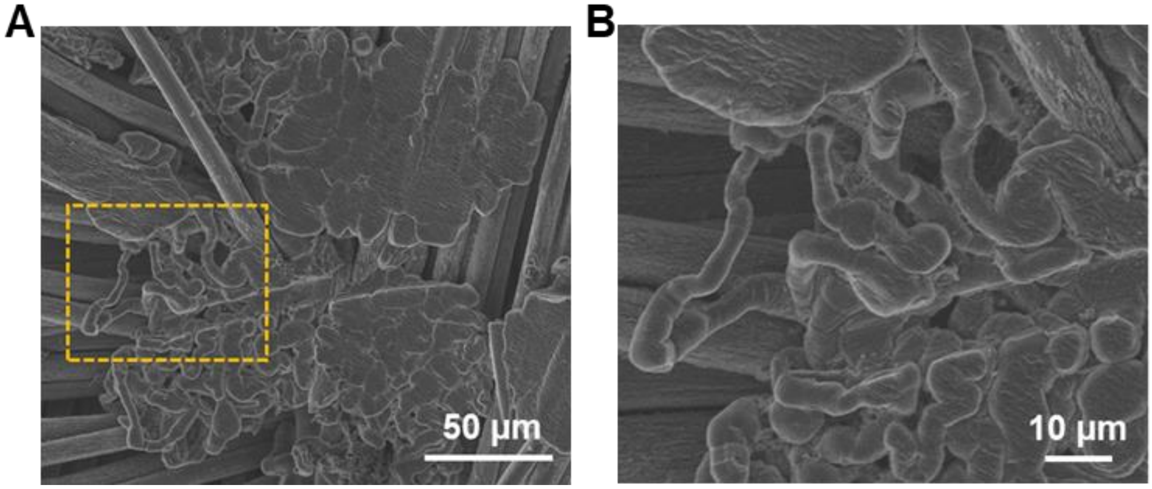


**Fig. S7. SEM images of Li-CF with 9 mAh cm$^{-2}$ plated Li.** **(B)** is the magnification of the yellow rectangle region in **(A)**.

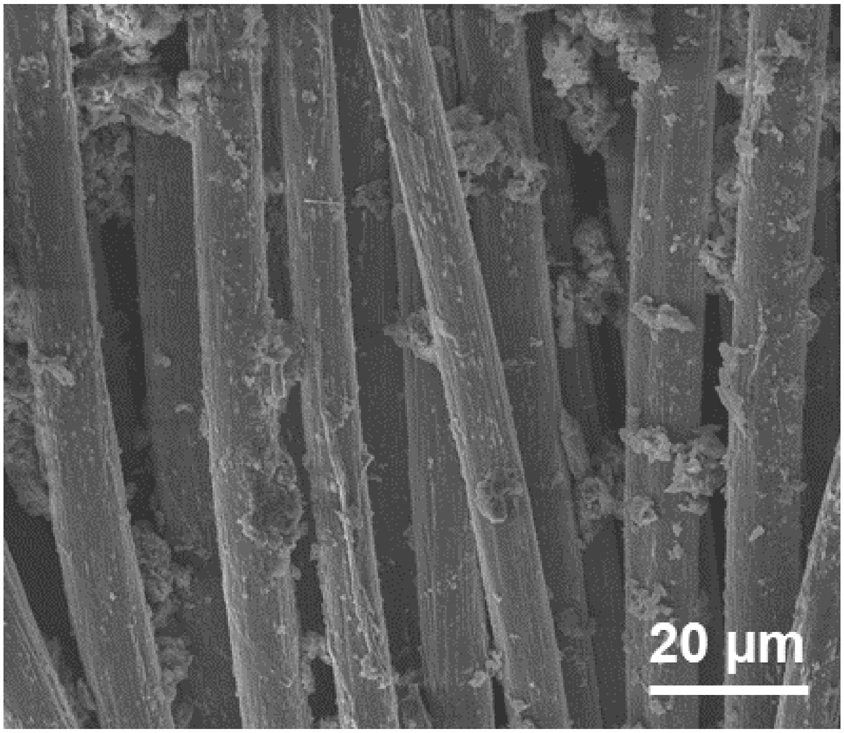


**Fig. S8**. **SEM image of Li-CF after Li stripping.**

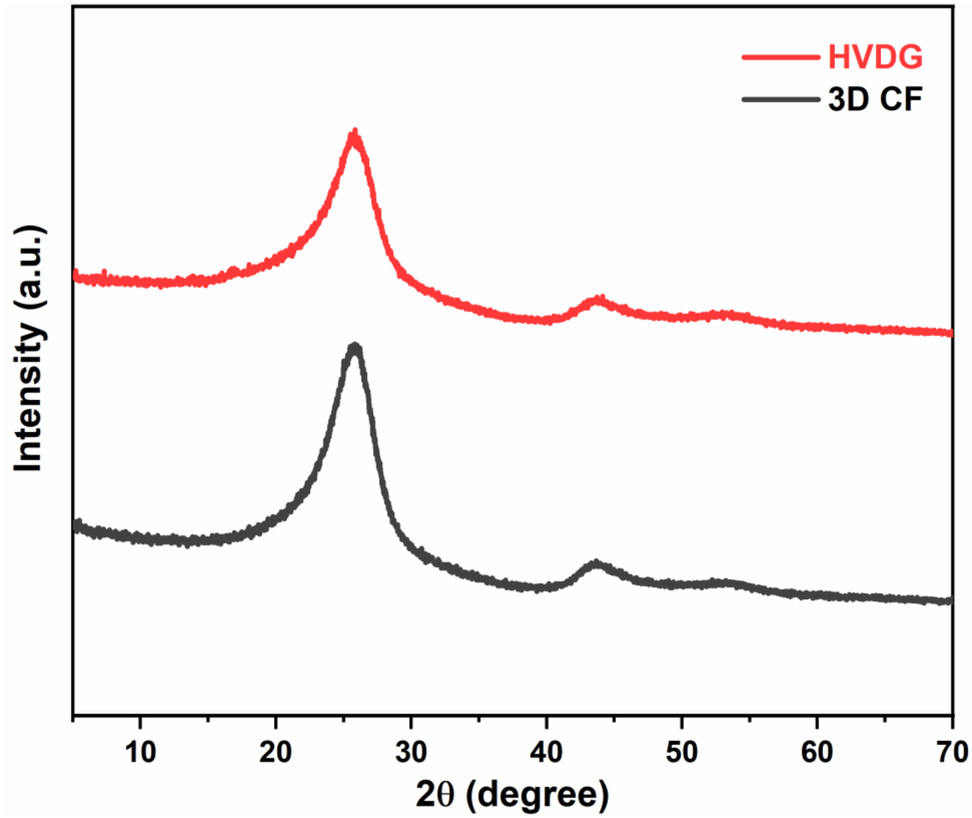

**Fig. S9**. **XRD patterns of 3D CF and HVDG.**

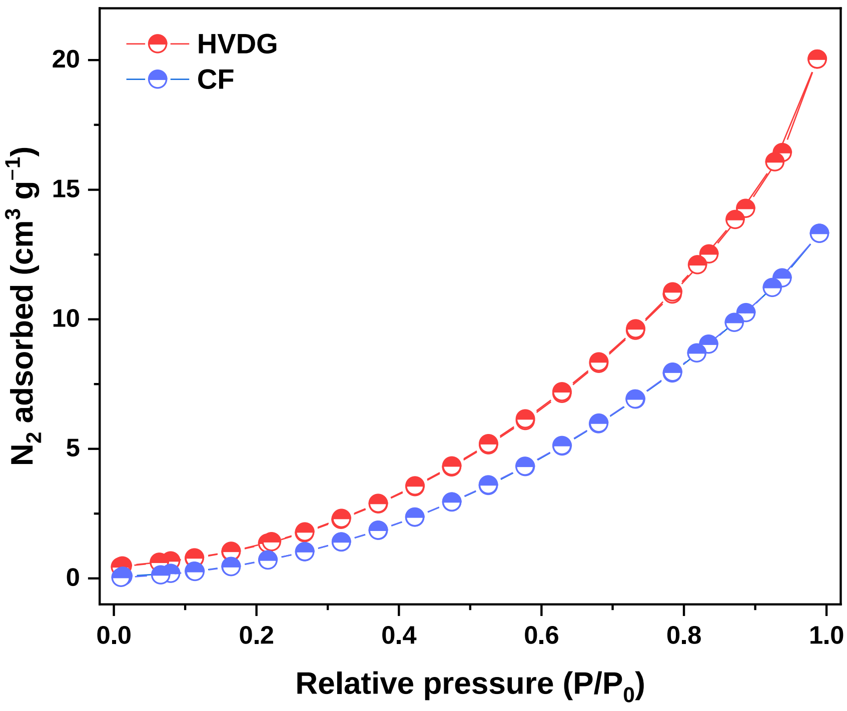


**Fig. S10 Nitrogen adsorption–desorption isotherms of HVDG and CF.**

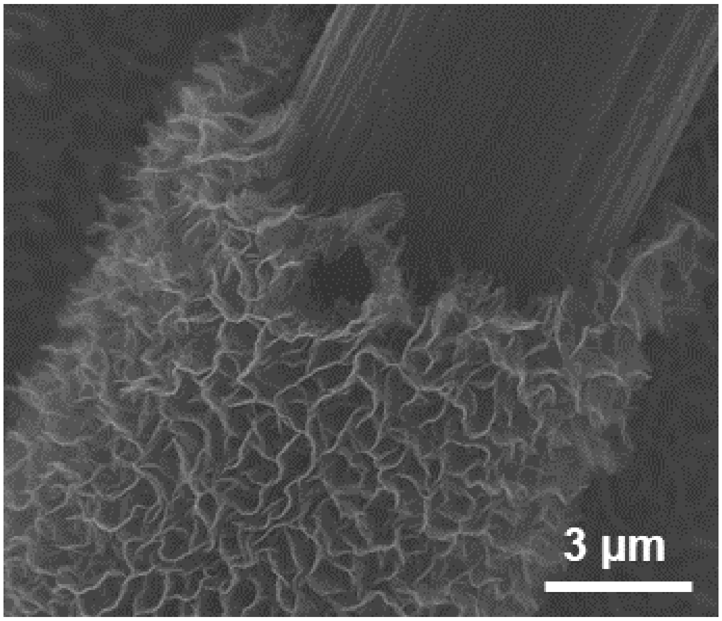


**Fig. S11. SEM image showing the thickness of the graphene arrays.**

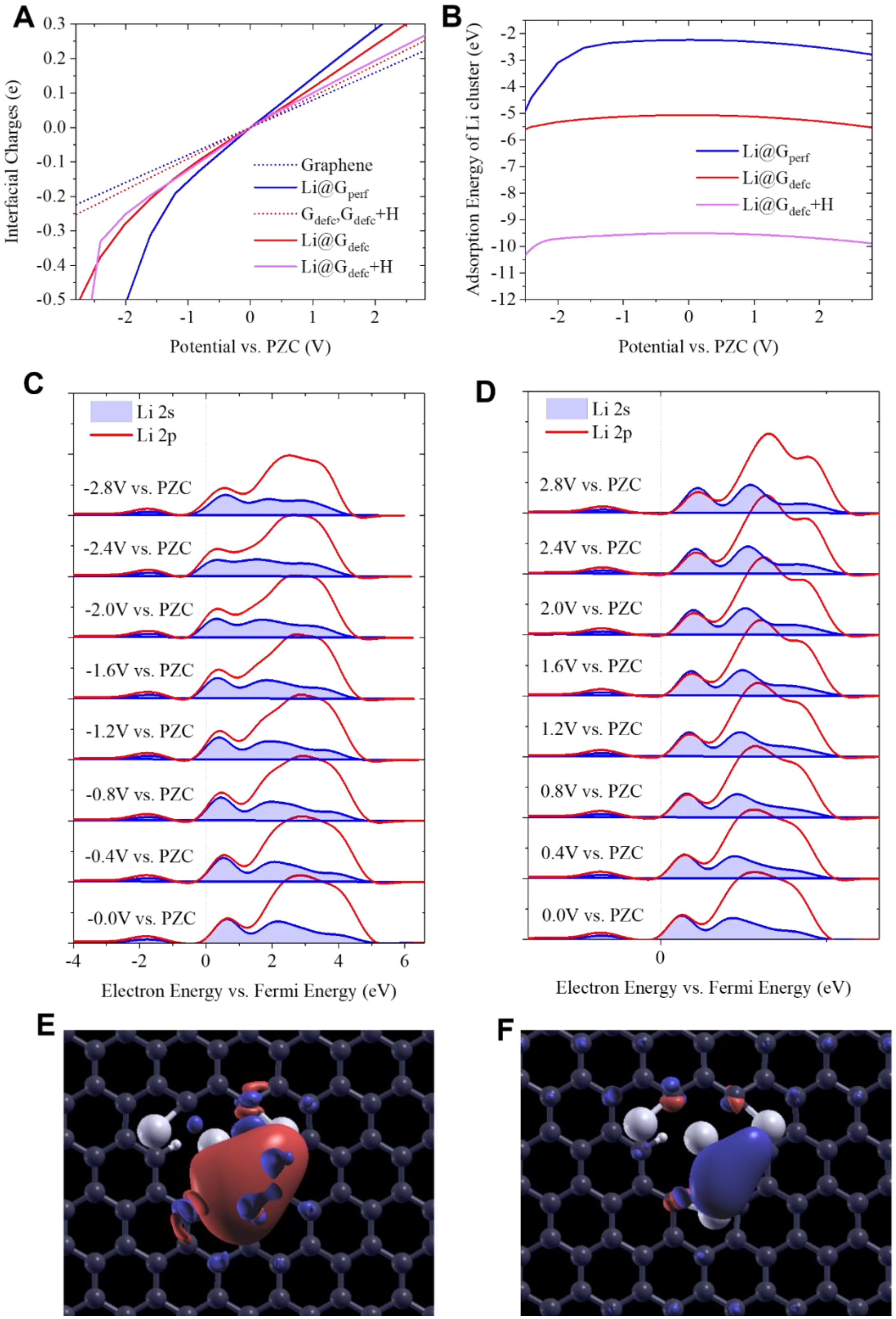
A
Interfacial Charges (e)
Potential vs. PZC (V)
Graphene
Li@G_perf
G_defc,G_defc+H
Li@G_defc
Li@G_defc+H
B
Adsorption Energy of Li cluster (eV)
Potential vs. PZC (V)
Li@G_perf
Li@G_defc
Li@G_defc+H
C
Li 2s
Li 2p
-2.8V vs. PZC
-2.4V vs. PZC
-2.0V vs. PZC
-1.6V vs. PZC
-1.2V vs. PZC
-0.8V vs. PZC
-0.4V vs. PZC
-0.0V vs. PZC
Electron Energy vs. Fermi Energy (eV)
D
Li 2s
Li 2p
2.8V vs. PZC
2.4V vs. PZC
2.0V vs. PZC
1.6V vs. PZC
1.2V vs. PZC
0.8V vs. PZC
0.4V vs. PZC
0.0V vs. PZC
Electron Energy vs. Fermi Energy (eV)
E
F

**Fig. S12. DFT analysis of the interactions between Li and different graphene matrixes. (A)** Interfacial charges as a function of applied potentials *vs.* potential of zero charge (PZC). Dotted lines represent models of perfect graphene ($G_{perf}$), defective graphene with vacancy ($G_{defc}$), and defective graphene with vacancy and hydrogenated carbon ($G_{defc+H}$). Solid lines show adsorption of a four-atom Li cluster on the three models accordingly. **(B)** Adsorption energy of the Li cluster on the three graphene models as a function of applied potentials *vs.* PZC. **(C-D)** Potential dependent partial density of states (pDOS) of one Li atom bonded to $G_{defc+H}$: **(C)** in the cathodic direction, **(D)** in the anodic direction. **(E-F)** Electron reservoir of **(E)** electron storage and **(F)** electron release at cathodic and anodic potentials, respectively. The red and blue colours show electron accumulation and depletion, respectively.

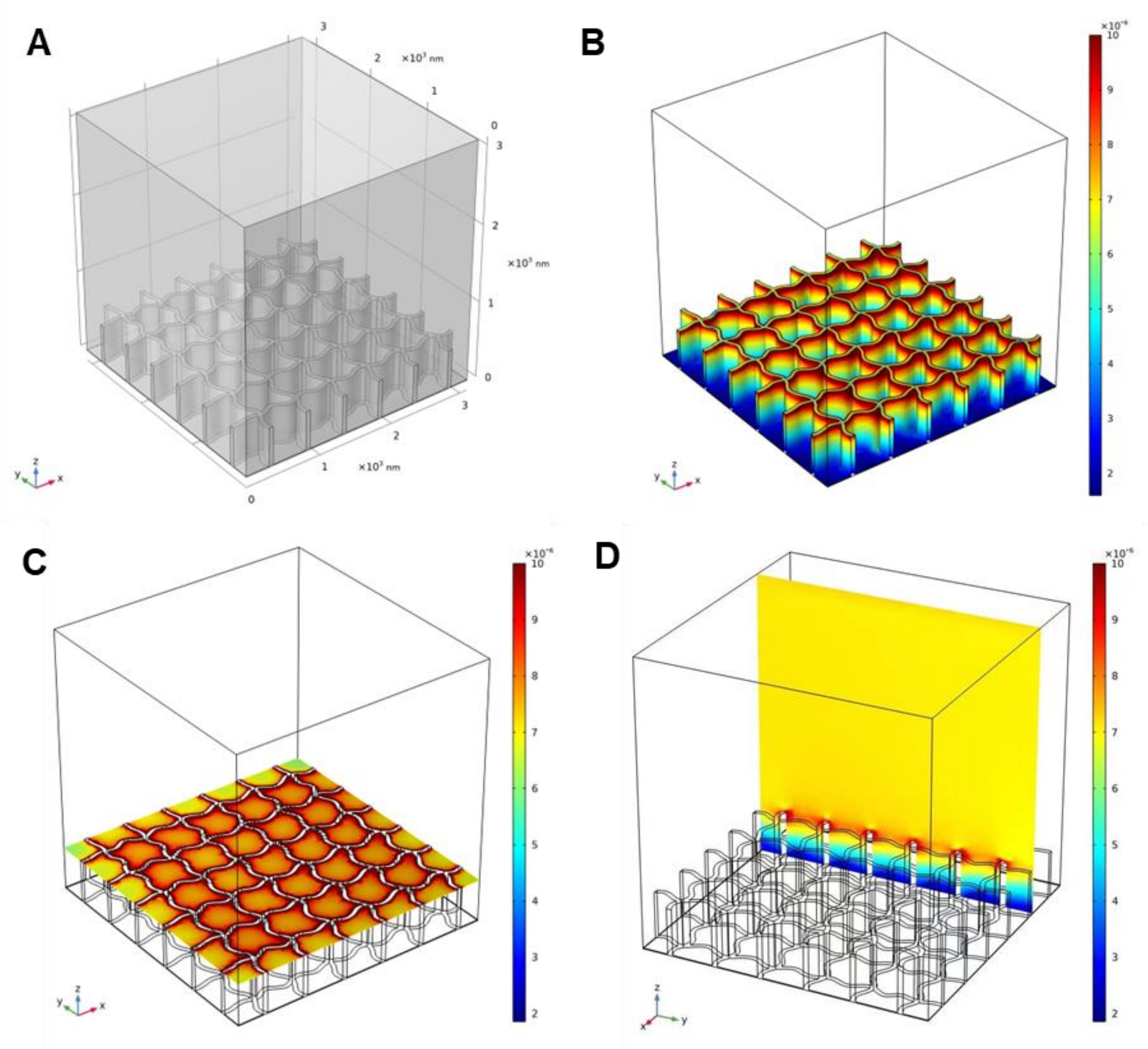


**Fig. S13. COMSOL simulations of Li$^{+}$ flux (current density) distribution on the surface of HVDG. (A)** The 3D model, **(B)** simulation results of the 3D model, **(C-D)** simulation results of 2D planes extracted from the 3D model. The unit is mol m$^{-2}$ s$^{-1}$.

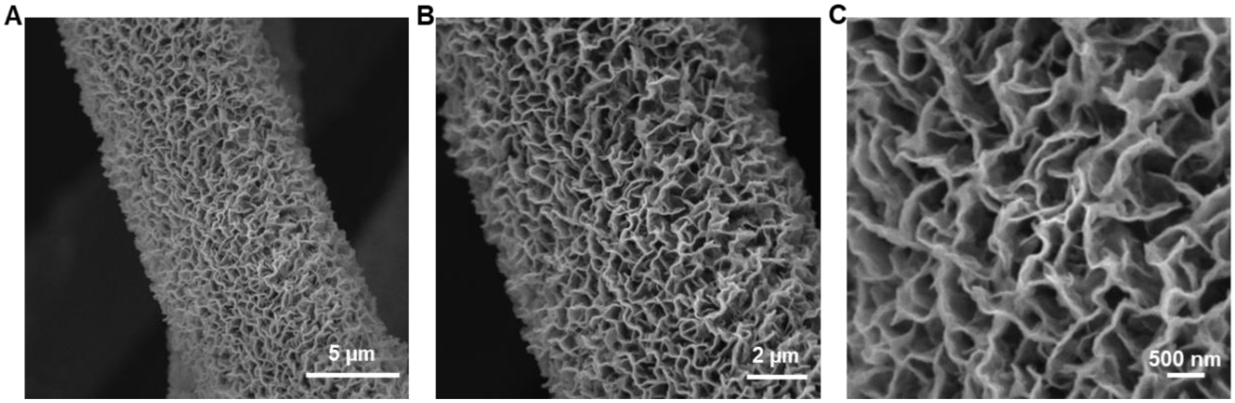


**Fig. S14. High-resolution SEM images of Li-HVDG after Li stripping.**

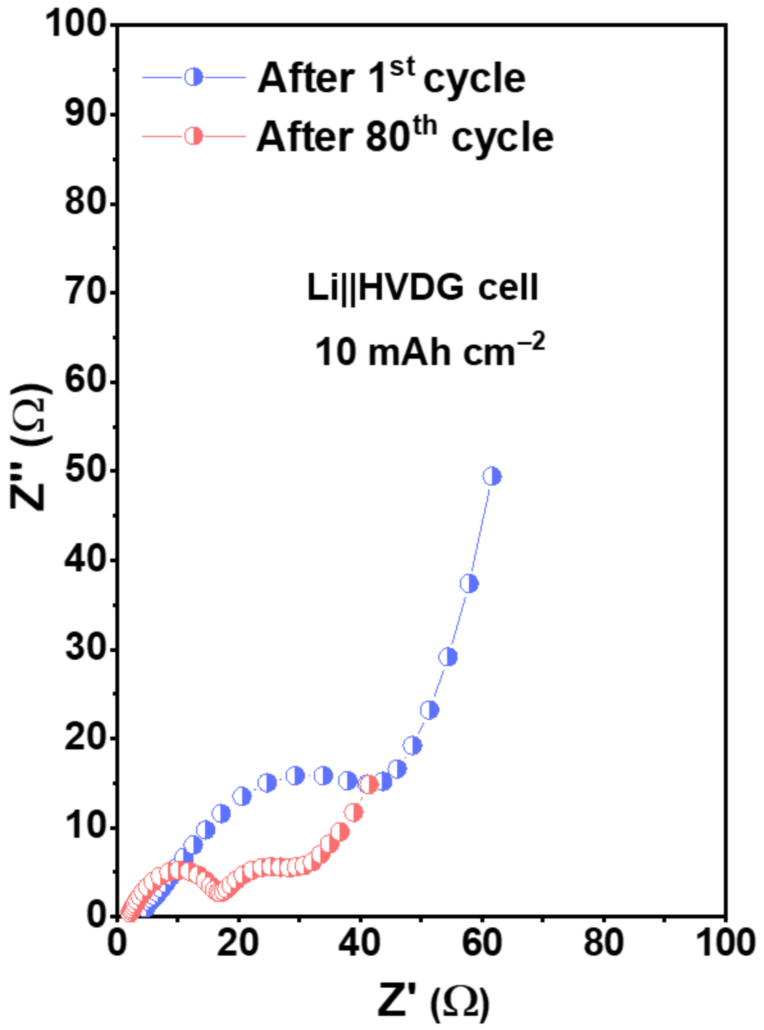


**Fig. S15. EIS profiles of the Li||HVDG cell after 1st and 80th cycles at an areal capacity of 10 mAh cm$^{-2}$.**

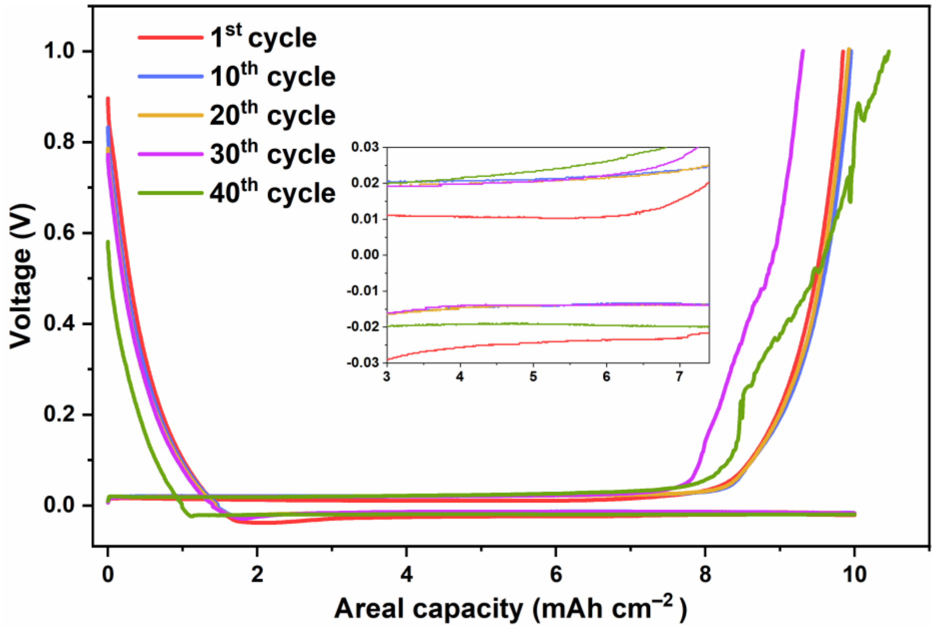


**Fig. S16. Voltage profiles of Li||CF cell with an areal capacity of 10 mAh cm$^{-2}$ at 1 mA cm$^{-2}$.**

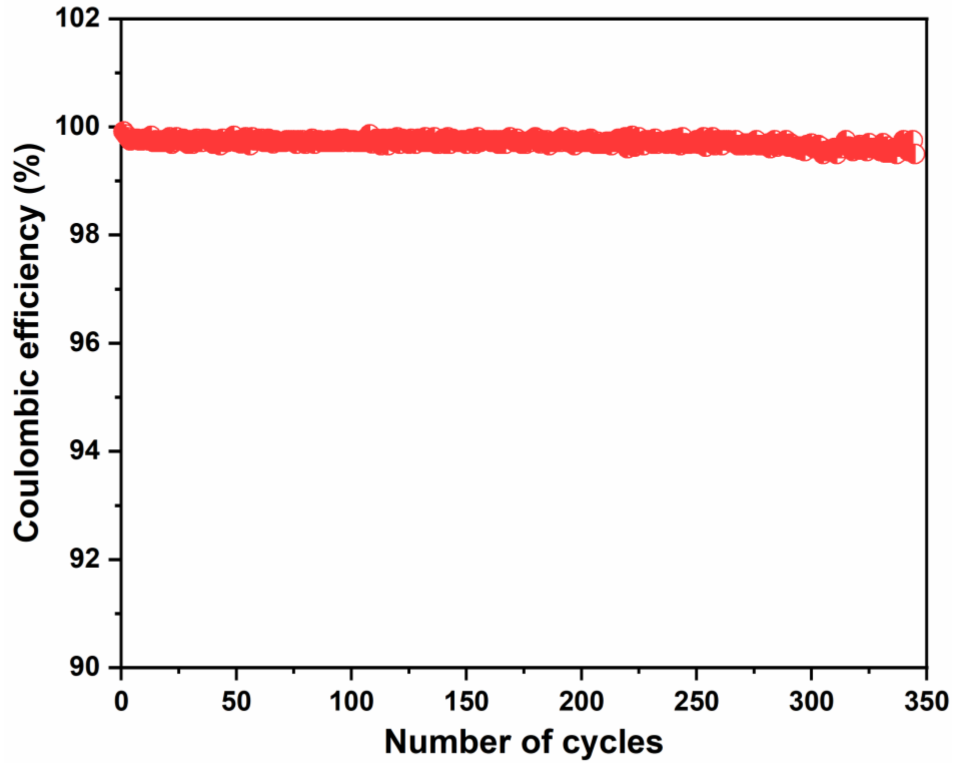


**Fig. S17. CE of Li||HVDG cell with a Li plating capacity of 1 mAh cm$^{-2}$ at 3 mA cm$^{-2}$.**

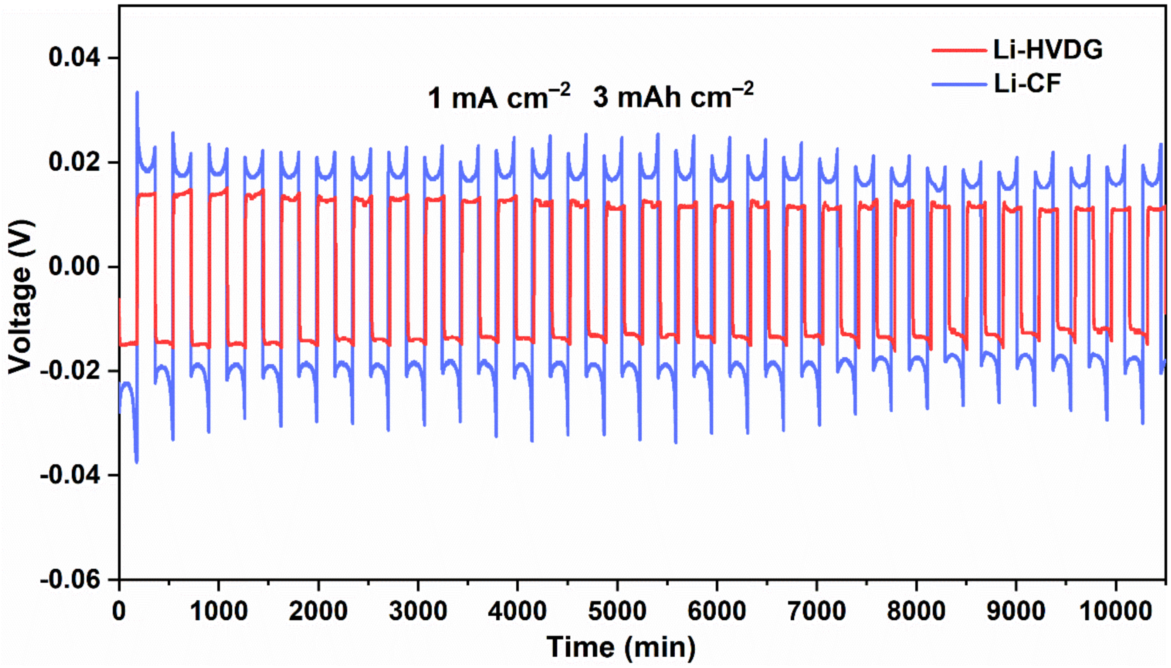


**Fig. S18. Galvanostatic cycling of symmetric Li-HVDG and Li-CF cells with a Li stripping/plating capacity of 3 mAh cm$^{-2}$ at 1 mA cm$^{-2}$.**

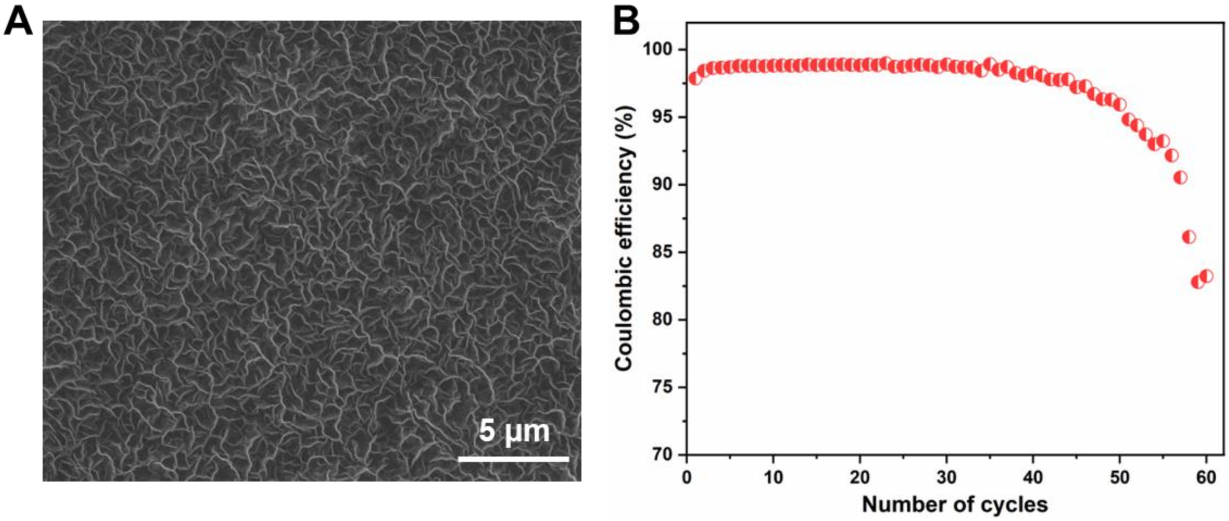


**Fig. S19. Vertical defective graphene arrays grown on Cu foil (VDG-Cu). (A)** SEM image. **(B)** CE of Li||VDG-Cu cell with a Li plating capacity of 3 mAh $cm^{-2}$ at 1 mA $cm^{-2}$.

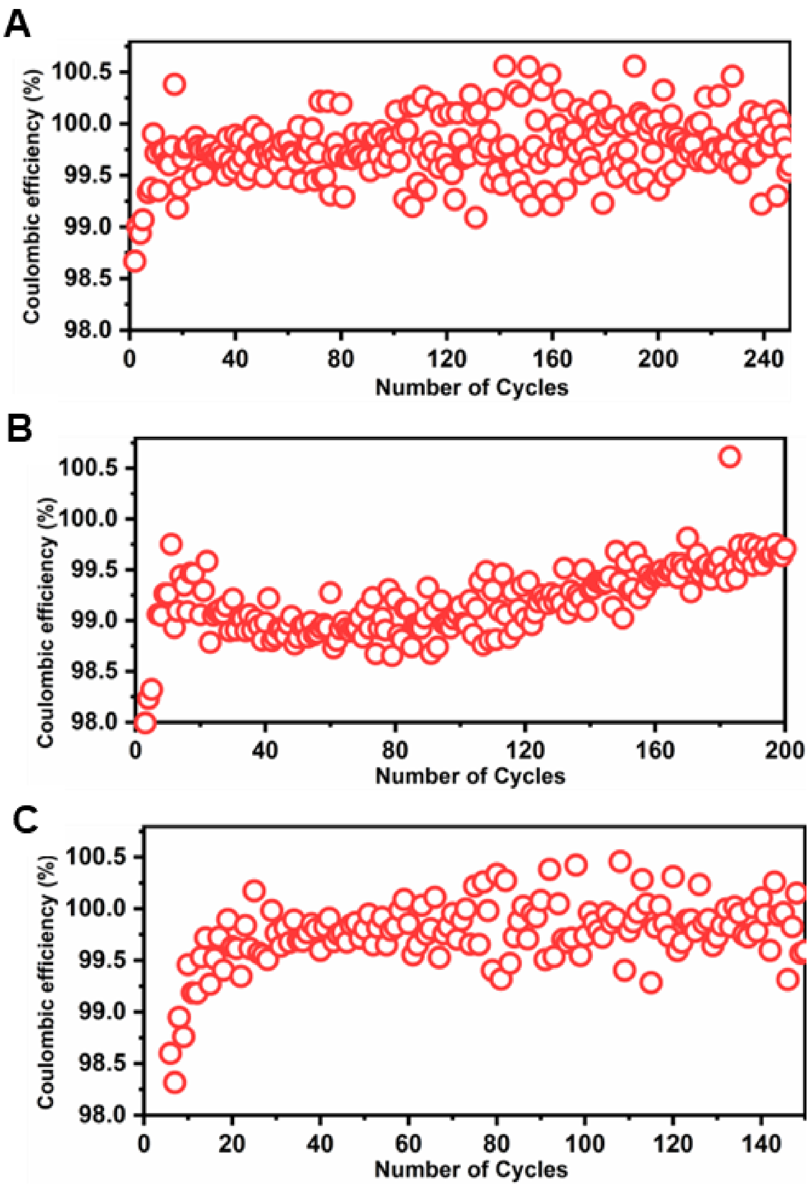


**Fig. S20. Detailed description of the Coulombic efficiencies of the HVDG-based Li metal full cells**. **(A)** Corresponding to Fig. 6A. **(B)** Corresponding to Fig. 6B. **(C)** Corresponding to Fig. 6C.

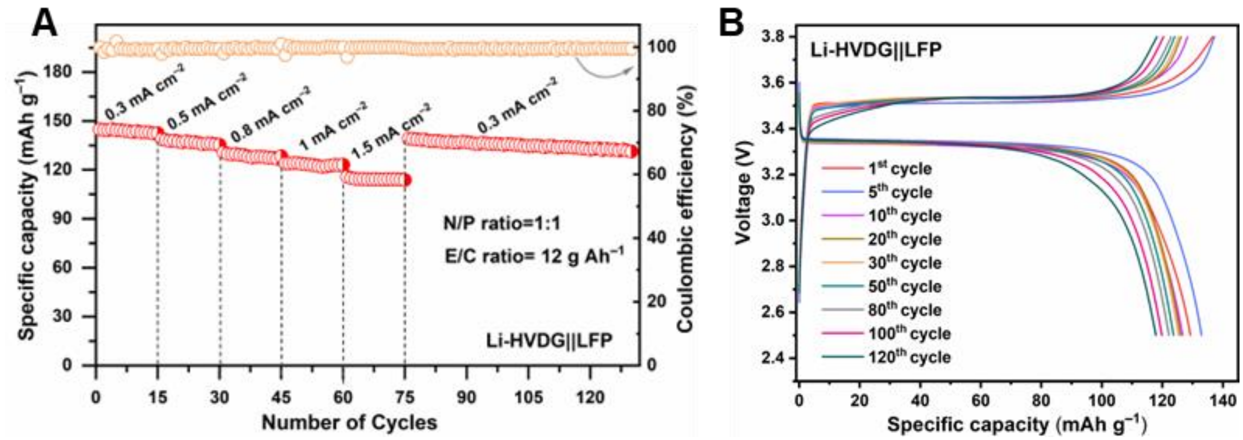


**Fig. S21. Rate performance of the Li-HVDG||LFP cell with a N/P ratio of 1:1 and E/C ratio of 12 g Ah$^{-1}$. (A)** Rate capability. **(B)** Corresponding charge/discharge profiles.

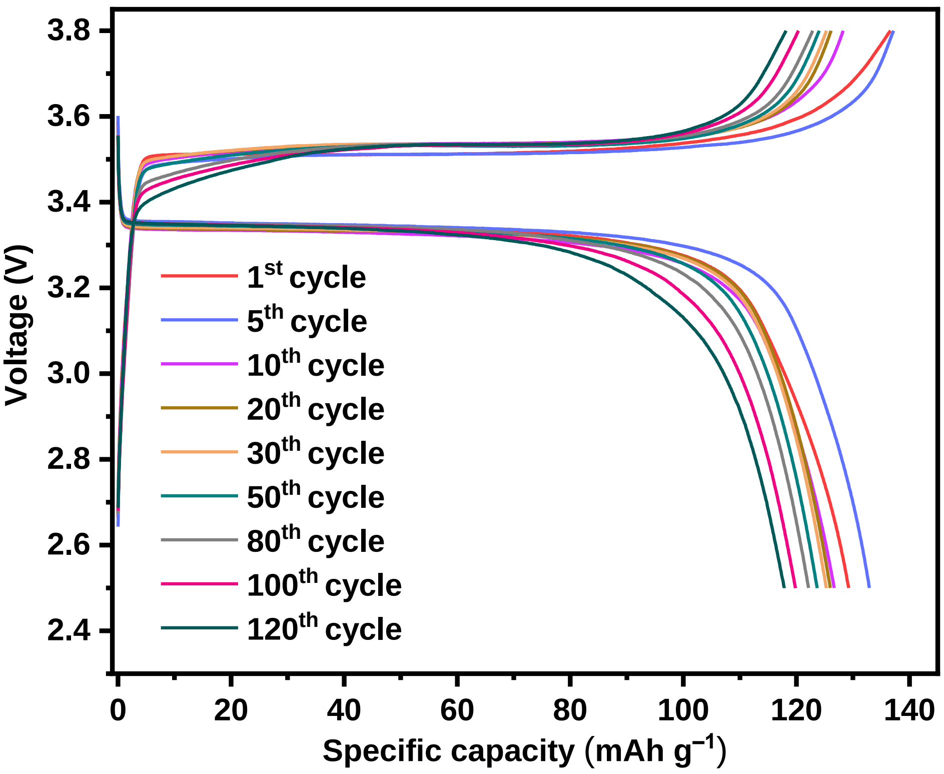


**Fig. S22. Charge/discharge profiles of Li-HVDG ||LFP cell at a current density of 0.15 mA cm$^{-2}$ with a N/P ratio of 1:1 and E/C ratio of 5 g Ah$^{-1}$.**

**Table S1** Details of the comparisons between the Li-HVDG||LFP cell in this work and recently reported Li metal full cells in literature.

| Ref. | Year | Cathode | Areal capacity (mAh $cm^{-2}$) | N/P ratio | E/C ratio (g $Ah^{-1}$) | Number of cycles | Capacity retention | Average CE | Current density (mA $cm^{-2}$) |
|---|---|---|---|---|---|---|---|---|---|
| This work | | LFP | 4.1 | 1 | 5 | 150 | 90% | 99.7% | 0.15 |
| *(14)* | 2019 | NCM622 | 3.8 | 2.6 | 3 | 200 | 86% | N/A | 0.38/1.3 |
| *(16)* | 2019 | NCM811 | 4.2 | 1.5 | 3 | 200 | 86% | N/A | 0.8/1.3 |
| *(19)* | 2019 | S | 1.2 | 3 | 8.3 | 65 | 73% | N/A | 1.68 |
| *(37)* | 2019 | NCM811 | 1.8 | 5 | 25 | 150 | 90% | 99.7% | 0.6 |
| *(38)* | 2020 | LFP | 3 | 3.5 | 6 | 100 | 60% | N/A | 1 |
| *(39)* | 2020 | NCM811 | 1.1 | 5.5 | 14 | 100 | 90% | 99.8% | 0.13 |
| *(40)* | 2020 | Mg/Ti-$LiNiO_2$ | 1.38 | 19.6 | 37 | 250 | 85% | N/A | 0.5/1.68 |
| *(41)* | 2020 | NCM811 | 3 | 1 | 8 | 50 | 86% | N/A | 1.44 |
| *(42)* | 2021 | LFP | 2.67 | 1.4 | 10 | 200 | 81% | N/A | 1.7 |
| *(43)* | 2021 | LFP | 2.7 | 2 | 6 | 300 | 94% | 99.5% | 2.55 |
| *(44)* | 2021 | LFP | 2.9 | 2.5 | 24 | 125 | 92% | N/A | N/A |
| *(45)* | 2021 | NCM811 | 3.8 | 2.4 | 7.5 | 150 | 82% | 99.5% | 1 |
| *(46)* | 2021 | NCM811 | 2 | 2 | 12 | 250 | 76% | N/A | 1 |
| *(47)* | 2021 | LFP | 1.8 | 1.7 | 33 | 500 | 73% | 99.8% | 1 |
| *(48)* | 2021 | LFP | 2 | 4 | 10.5 | 140 | 98% | N/A | 0.44 |
| *(49)* | 2021 | LFP | 2 | 3.5 | 20 | 100 | 74% | N/A | 0.2 |
| *(50)* | 2022 | LFP | 3.2 | 3 | 5 | 270 | 93% | N/A | 3.2 |
| *(51)* | 2022 | NCM622 | 3.8 | 10.5 | 21 | 100 | 97% | 99.2% | 0.88 |
| *(52)* | 2022 | LFP | 3.14 | 1.3 | 19.7 | 370 | 100% | N/A | 1.7 |
